# “Rewiring brain immunity: targeting microglial metabolism for neuroprotection in neurodegenerative disorders”

**DOI:** 10.1007/s11011-025-01739-y

**Published:** 2025-11-25

**Authors:** Mustafa M. Shokr

**Affiliations:** Department of Pharmacology and Toxicology, Faculty of Pharmacy, Sinai University – Arish Branch, Arish, 45511 Egypt

**Keywords:** Microglial metabolism, Neuroinflammation, Metabolic reprogramming, Neurodegeneration

## Abstract

Neuroinflammation, a pervasive hallmark in many neurological and neuropsychiatric diseases, is largely dictated by the functional phenotypic dynamics of microglia, the immune system of the brain. Recent data illustrate that these phenotypic changes, from neuroprotective scavenging to neurotoxic pro-inflammatory effects, are intrinsically regulated by microglial metabolic repolarization. This review synthesizes understanding of discrete microglial metabolic phenotypes like the glycolytic reliance of pro-inflammatory (M1-like) microglia and the oxidative phosphorylation/fatty acid oxidation bias of anti-inflammatory/resolving (M2-like) microglia. We discuss how central metabolic sensors like AMPK, mTOR, and HIF-1α oversee these metabolic shifts in response to disease-targeted pathologies in Alzheimer’s, Parkinson’s, Multiple Sclerosis, ischemic stroke, and traumatic brain injury. Moreover, we review innovative therapeutic strategies directed toward microglial metabolism, involving pharmacological modulators (e.g., metformin, rapamycin, and ketone bodies), nutritional interventions (e.g., ketogenic diets), and modulation of gut microbiota. By tightly specific re-tuning of microglial cells’ bioenergetics, these approaches enable unprecedented opportunities to counteract neuroinflammation, enhance pathological clearance, and induce neuroprotection, paving the way for a new generation of disease-modifying therapies of neurodegenerative disorders.

## Introduction

From acute injuries like stroke and traumatic brain injury (TBI) to chronic neurodegenerative diseases like Alzheimer’s disease (AD), Parkinson’s disease (PD), and multiple sclerosis (MS), as well as major depressive disorder and autism spectrum disorder, neuroinflammation, a dynamic and complex immune response in the central nervous system (CNS), is a ubiquitous hallmark across the spectrum of neurological and neuropsychiatric illness (Abulaban et al. 2025a). This complex inflammatory process, which is primarily controlled by the brain’s resident macrophages, or microglia, is anything but an innocent bystander. It can either aid in healing, protective tissue repair, and pathogen removal, or, if left unchecked and prolonged, it can cause progressive neuronal damage and functional decline (Abulaban et al. 2025a). Microglia are highly plastic cells that constantly examine their microenvironment in a highly branched, “sleeping” condition, carrying out vital homeostatic activities necessary for preserving brain health. They comprise roughly 5–10% of all glial cells in the CNS (Nimmerjahn et al. 2005; Rajab et al. 2025). These essential functions include trophic support delivery to neurons, clearing away cellular debris and abnormally folded proteins (such as alpha-synuclein in PD and amyloid-beta in AD), regulating neuronal excitability, and pruning synapses during development and plasticity (Miao et al. 2023a; Badawi et al. 2024; Khowdiary et al. 2025). But when faced with polyvalent pathogenic stimuli, such as infections, aggregated proteins, ischemia, or damage, microglia quickly activate and change their shape and functional phenotype (Amruta et al. 2020). The ability of microglia to switch between a pathologic, pro-inflammatory state (M1-like, which releases neurotoxic mediators like reactive oxygen species (ROS), nitric oxide, and pro-inflammatory cytokines TNF-α) and a normal, pro-resolving state (often referred to as M2-like, consisting of anti-inflammatory cytokines, tissue repair, and enhanced phagocytosis) is a point of separation (Alrouji et al. 2023).

The term metabolic reprogramming has been coined as a determining factor of microglial phenotype and function because of new evidence that clearly shows that this significant conversion from healthy to pathologic microglial states is established largely by their metabolic programming (Gim? nez-Cassina and Cuezva 2022). Like peripheral immune cells, microglia are remarkably metabolically flexible, altering their energy generation routes to suit the demands of their functional state. To maintain their basal monitoring function, homeostatic microglia primarily use efficient oxidative phosphorylation (OXPHOS), which uses fatty acids and glucose as substrates to produce ATP (Xu et al. 2023). However, even in oxygen-containing environments, microglia quickly reroute their metabolism in response to inflammatory stimuli, often shifting toward aerobic glycolysis (Mehla and Singh 2019). Quick ATP is provided by this glycolytic burst to support immediate effector functions, such as the generation and release of pro-inflammatory cytokines. However, it can also result in the buildup of certain metabolic byproducts, such as succinate, which intensify inflammation through the NLRP3 inflammasome and other pathways (Anderson et al. 2023). On the other hand, microglia are likely to reactivate OXPHOS and depend more on fatty acid oxidation (FAO) for long-term functions such as phagocytosis, tissue repair, and the generation of anti-inflammatory mediators (Muzio et al. 2021). A prolonged reliance on ineffective or dysregulated metabolic states can prolong chronic inflammation and impair the microglial capacity for resolution and repair, which directly contributes to the etiology of neurodegenerative diseases. This is because metabolic pathways and their functional outcomes are intricately interdependent (Al-Kuraishy et al. 2023).

Alpha-synuclein aggregation and dopaminergic neuronal vulnerability may be facilitated by impaired metabolism in microglia in Aβ (Alrouji et al. 2024a; Abulaban et al. 2025b). Similarly, the demyelination/remyelination balance in MS is regulated by metabolic changes in migratory macrophages and microglia (Al-Kuraishy et al. 2024c). Thus, comprehending the subtleties of microglial metabolic reprogramming is not merely a theoretical undertaking; rather, it represents a novel area of CNS research with unheard-of therapeutic potential (Al-Kuraishy et al. 2024c). To lay the groundwork for new precision medicine approaches in neurology, this review aims to compile the most recent research on these crucial microglial metabolic shifts in a range of neurodegenerative diseases, examine the molecular basis for these shifts, and offer insights into the potential therapeutic benefits of modifying microglial metabolism to coordinate neuroinflammation and ultimately stop or reverse the progression of neurodegenerative diseases (Al-Kuraishy et al. 2024a).

Recent human single-cell and spatial transcriptomics reveal microglia within a continuum of functional states, such as the Disease-Associated Microglia of AD or the Lipid-Droplet-Accumulating Microglia (LDAM) of aging and resolution failure (Marschallinger et al. 2020). Metabolic reprogramming is the driving force along this continuum: the traditional M1-like pole is matched with the states of high glycolytic reliance and HIF-1α signaling necessitated for the rapid, pro-inflammatory bursts. In comparison, homeostatic and pro-resolving (the former M2-like pole) states are governed by a metabolic switch towards sustained OXPHOS and Fatty Acid Oxidation (FAO), which are crucial for energy-intensive functions like phagocytosis and repair of tissue, that tend to be regulated by the AMPK/PGC-1α pathway (Marschallinger et al. 2020). Notably, metabolic dysregulation, i.e., compromised FAO or mitochondrial dysfunction, has a direct impact on pathological conditions like LDAM formation, wherein there is lipoprotein accumulation due to failed clearance and resulting in chronic neuroinflammation, signaling that the balance between catabolic and anabolic pathways dictates microglial fate across the entire spectrum of disease (Cao et al. 2025a).

## Methodology

### Literature search strategy

A comprehensive search was conducted in PubMed, Scopus, and Web of Science up to July 2025, using combinations of keywords: “Alzheimer’s disease,” “Parkinsonism,” “Microglia, “and “Multiple sclerosis,”. Reference lists of relevant articles were manually screened for additional studies.

### Inclusion and exclusion criteria

The inclusion criteria for relevant clinical trials and human studies are based on patients with established neurodegenerative disorders, Alzheimer’s, Parkinson’s, and Multiple Sclerosis, or acute CNS pathologies involving ischemic stroke, where microglial-mediated neuroinflammation represents a key pathomechanism. Included populations must be amenable to therapeutic intervention through metabolic reprogramming using pharmacological modulators (e.g., metformin, rapamycin), nutritional intervention (e.g., ketogenic diets), or modulation of the gut microbiota (e.g., probiotics). Exclusion criteria are designed to minimize confounding metabolic variables and typically exclude patients with severe and uncontrolled systemic inflammatory or metabolic comorbidities, acute active infections, or concomitant treatment with medications (such as broad-spectrum antibiotics) that inhibit the desired effects on microglial or gut metabolic pathways.

### Data extraction and synthesis

Synthesis and extraction of data for this manuscript will strictly involve the extraction and collection of data regarding microglial metabolic reprogramming from peer-reviewed in vitro, in vivo, and clinical studies. We will pull information predominantly regarding the function of AMPK, mTOR, and HIF-1α as the central regulators, metabolic phenotypes (glycolysis vs. OXPHOS), and functional repercussions (pro- vs. anti-inflammatory marker expression) from such alterations across neurodegenerative diseases. Data regarding therapy, including the efficacy of pharmacological modulators, nutritional therapy, and gut microbiome modulation, will be incorporated to evaluate the impact on microglial function and neuroinflammation. Synthesis will be placed on identifying universal mechanisms, showing translational challenges, and guiding the direction of the future to single-cell multi-omics and precision therapies.

### Study selection and PRISMA flow

Study selection will adhere to PRISMA standards and employ a systematic search of principal databases (e.g., PubMed, Scopus) through keyword-specific to microglial metabolism (AMPK, mTOR, HIF-1α), neuroinflammation, and neurodegenerative diseases. The initial search will identify all relevant preclinical (in vitro and in vivo) and human clinical trials on the modulation of microglial metabolic states. Title and abstract screening and full-text review, completed independently by two reviewers, will be utilized to include studies that report specifically on microglial metabolic fate (glycolysis vs. OXPHOS) and the efficacy of the targeted metabolic or gut-microbiome interventions.

## Differences between rodent and human microglia

A major obstacle to bench-to-bedside translation of microglial metabolic studies is the deep species difference between commonly used rodent models and human physiology. Human microglia, particularly those involved in neurodegeneration, exhibit unique patterns of gene expression, such as the exclusive disease-associated microglia signature, that do not precisely translate to the M1/M2 metabolic phenotypes largely characterized in murine cells (Fornari Laurindo et al. 2024).

These functional and transcriptional distinctions delimit the straightforward extrapolation of metabolic pathways and therapeutic response from mice. Furthermore, a significant technical challenge is the lack of strong, non-invasive biomarkers directly reflecting the metabolic status (e.g., glycolytic flux, FAO activity, or NAD+/NADH ratio) of CNS microglia in vivo in patients (Fornari Laurindo et al. 2024). To address this gap, translational strategies must prioritize surrogate marker and imaging readout validation. This includes rigorously ascertaining if peripheral markers, i.e., specific cytokines or circulating metabolites, or less-invasive CSF metabolites (e.g., lactate or kynurenine pathway intermediates), reliably correlate with ex vivo metabolic profiles of human iPSC-derived microglia or human post-mortem tissue.

In tandem, non-invasive neuroimaging modalities, particularly PET scanning using probes of glucose metabolism (e.g., [18 F] FDG uptake as a surrogate of glycolysis) or using radiolabeled TSPO ligands (which mark microglial activation/mitochondrial density), must be developed and rigorously correlated with functional and clinical endpoints (Zhang et al. 2020). Such a multi-faceted validation approach, integrating peripheral, CSF, and neuroimaging data, is required to derive clinically meaningful readouts for precision microglial metabolic therapies (Zhang et al. 2020).

## Metabolic regulators and sensors of microglia

Their metabolic status, which is controlled by an extremely complex network of vital metabolic sensors and regulators, richly orchestrates the highly coordinated immune cell activity dance, especially in the dynamic microenvironment of the CNS (Gim? nez-Cassina and Cuezva 2022). These molecular arbiters continuously monitor the cellular energy state, food availability, and redox balance. They then use this information to generate precise metabolic responses that determine the phenotypic and functional output of immune cells. AMP-activated protein kinase (AMPK), mammalian target of rapamycin (mTOR), and hypoxia-inducible factor 1 alpha (HIF-1α) are some of the most interconnected nodes in this regulatory circuit. They all have different but related functions in coordinating the metabolic reprogramming required for immune cell function, and particularly microglial function in both health and disease (Gim? nez-Cassina and Cuezva 2022).

When the AMP: ATP ratio rises, the evolutionarily conserved serine/threonine kinase known as AMPK, the cell’s primary energy sensor, becomes acutely active, indicating a lack of energy in the cell (Hardie 2008). In addition to this “canonical” activation by energy stress (such as glucose deprivation, hypoxia, or oxidative stress), AMPK can also be activated by lysosomal stress, calcium entry (via CaMKKbeta), and several pharmaceutical drugs, including AICAR and metformin (Yibcharoenporn et al. 2025). When activated, AMPK starts a broad program that aims to restore energy equilibrium by preventing energy-wasting anabolic processes and promoting ATP-producing catabolic processes. Its downstream targets are numerous and varied: AMPK directly phosphorylates and activates several important enzymes involved in mitochondrial biogenesis (through PGC-1alpha), fatty acid oxidation (through inhibition and phosphorylation of acetyl-CoA carboxylase, which eliminates the inhibition of carnitine palmitoyl transferase 1 (CPT1), and glucose uptake (e.g., by causing GLUT1 translocation to the plasma membrane) (Mihaylova and Shaw 2011). At the same time, AMPK inhibits anabolic activities like cholesterol synthesis, lipid synthesis, and protein synthesis (by inhibiting mTORC1, which is explained below, and phosphorylating *eIF2alpha*) (Al-Kuraishy et al. 2025). By promoting long-lasting processes like phagocytosis and debris removal and promoting effective energy synthesis through OXPHOS and FAO, AMPK activation in immune cells seems to favor a resting or anti-inflammatory/pro-resolving phenotype. For example, activation of AMPK in macrophages and microglia can promote anti-inflammatory mediators and decrease the manufacture of pro-inflammatory cytokines (e.g., TNF-α, IL-1β) by inhibiting NF-κB and inflammasome activation (O’Neill and Grahame Hardie 2013). For this reason, AMPK is a crucial therapeutic target in the fight against chronic neuroinflammation.

**Fig. 1:**
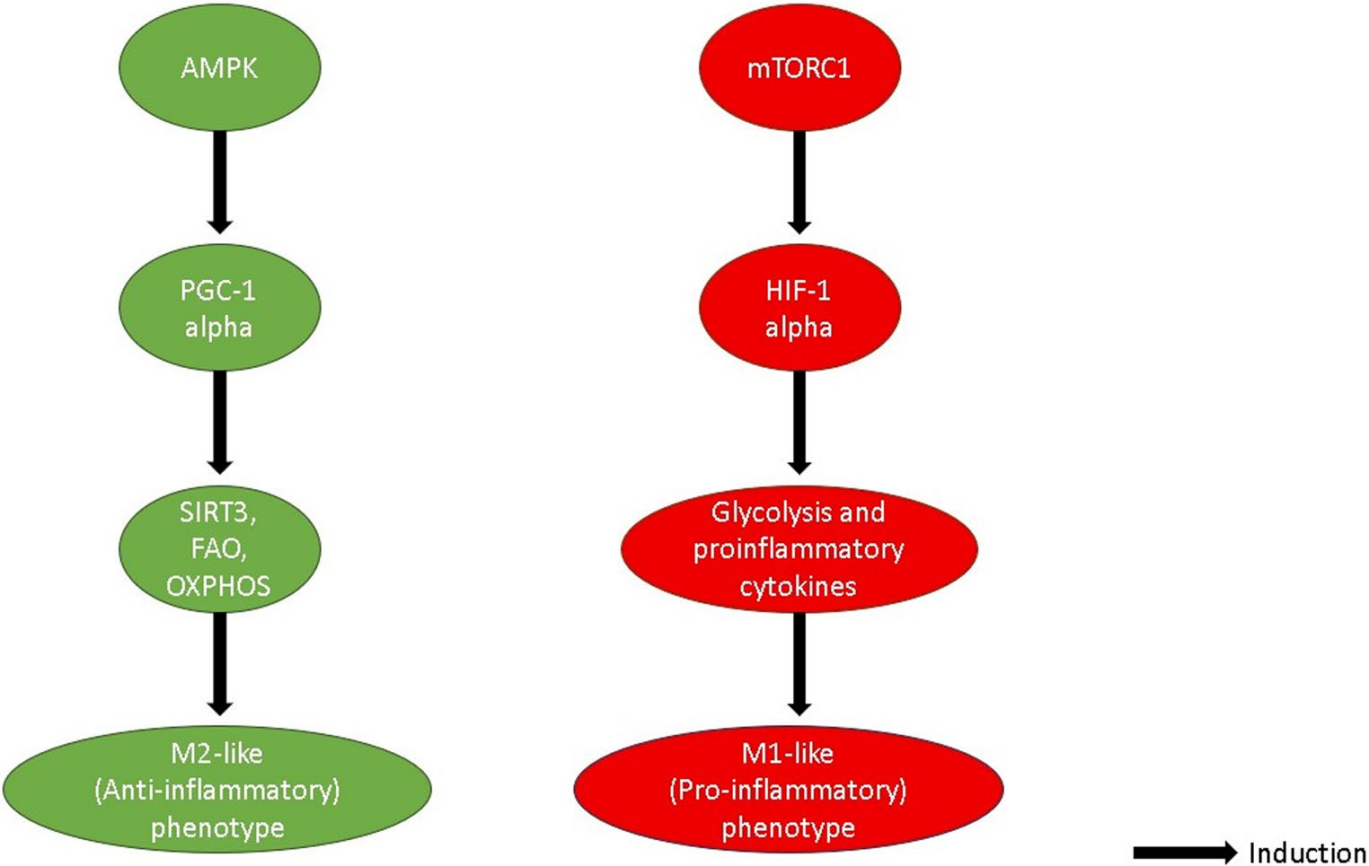
Opposing Metabolic Pathways Regulate Microglial Phenotype. This figure illustrates the two dominant central control pathways that play significant roles in the polarization and activity of microglial cells. On the left, the green pathway is the anti-inflammatory (M2-like) microglial phenotype. This pathway is activated when AMPK (AMP-activated protein kinase) is activated, followed by the activation of PGC-1α (Peroxisome proliferator-activated receptor-gamma coactivator 1-alpha). PGC-1α then upregulates genes towards the promotion of SIRT3 (Sirtuin 3), FAO (fatty acid oxidation) and OXPHOS (oxidative phosphorylation) such that the biogenesis and activity of the mitochondria are increased. Overall, the end-point is the creation of an M2-like (anti-inflammatory) phenotype, characterized by the resolution of the inflammation and the healing of the tissues. Conversely, the right-hand-side, the red pathway, is the pro-inflammatory (M1-like) microglial phenotype. This pathway is driven by the activation and promotion of the mTORC1 (mammalian target of rapamycin complex 1) such that the activation and stabilization of the transcription factor HIF-1α (Hypoxia-inducible factor 1-alpha) is brought about. HIF-1α then upregulates the glycolysis as well as the production of the pro-inflammatory cytokines such that there is the creation of the M1-like (pro-inflammatory) phenotype, characterized by active inflammation and the induction of the destruction of tissues. Solid black arrows indicate induction/activation along each pathway

mTOR is a master nutrient and growth factor sensor that combines data from amino acids, glucose, growth factors (including IGF-1), and cellular energy state to control cell growth, proliferation, and metabolism (Saxton and Sabatini 2017; Liu and Sabatini 2020). This stands in stark contrast to AMPK’s energy-sensing role [Figure 1]**.** Two separate multiprotein complexes, mTOR Complex 1 (mTORC1) and mTOR Complex 2 (mTORC2), each of which has its components, upstream activators, and downstream effectors, contain mTOR as a component (Laplante and Sabatini 2012). The most well-studied complex, mTORC1, is triggered by growth factors (via the PI3K/Akt pathway), an excess of energy, and adequate nutritional supply, particularly amino acids. S6 Kinase 1 (S6K1) and the eukaryotic initiation factor 4E-binding protein 1 (4E-BP1) are the main downstream targets; their phosphorylation stimulates protein synthesis and cell division (Mafi et al. 2022). A powerful inhibitor of autophagy, the cellular process of self-digestion essential for eliminating damaged organelles and abnormally folded proteins, mTORC1 also plays a significant role in the production of nucleotides and lipids (Al-Kuraishy et al. 2024c). Strongly proliferative and metabolic states in immune cells, such as the pro-inflammatory (M1-like) cell state, are characterized by robust mTORC1 activation, which promotes the quick production of inflammatory mediators (Jung et al. 2025). Growth hormones (insulin, IGF-1) activate the less well-known mTORC2, which phosphorylates Akt (Ser473), *PKCα*, and other kinases to control cell survival, cytoskeletal structure, and metabolism (Dos et al. 2004).

A master regulator of the cellular hypoxic response (decreased oxygen), hypoxia-inducible factor 1-alpha (HIF-1α) is also essential for metabolic reprogramming in normoxic inflammation (Semenza 2012). Prolyl hydroxylase domain (PHD) enzymes quickly hydroxylate HIF-1α in normoxia, causing the VHL E3 ligase complex to ubiquitinate it and then degrade it via proteasomes. However, PHD activity is inhibited in response to hypoxia or inflammatory stimuli (such as ROS, LPS, or pro-inflammatory cytokines), stabilizing and allowing HIF-1α to migrate to the nucleus (Alrouji et al. 2023). HIF-1α and HIF-1β heterodimerize within the nucleus and bind to hypoxia-response elements in target gene promoters to promote the expression of glycolytic enzymes required for aerobic glycolysis, such as lactate dehydrogenase A, phosphofructokinase, hexokinases, and glucose transporters (GLUT1, GLUT3) (Qannita et al. 2024). By causing pyruvate dehydrogenase kinase 1 to phosphorylate and inhibit pyruvate dehydrogenase (PDH), HIF-1α simultaneously suppresses OXPHOS and diverts pyruvate away from the mitochondria (Papandreou et al. 2006). For immune cells functioning in low-oxygen or highly inflammatory environments, this metabolic reprogramming to glycolysis offers a quick, oxygen-independent source of energy. The pro-inflammatory activation property of HIF-1α stabilization in microglia permits the glycolytic phenotype, which maintains the production of inflammatory cytokines and contributes to neurotoxicity (Papandreou et al. 2006).

The complex interactions and cross-talk between AMPK, mTOR, and HIF-1α create a tightly controlled network that promotes suitable metabolic adaptation. As an example, the AMPK-mTORC1 pathway: AMPK stimulates and phosphorylates TSC2 and raptor, two mTORC1 subunits that both suppress mTORC1 activity (Abulaban et al. 2025a). Thus, mTORC1 inhibition suppresses anabolic pathways in a low-energy state (AMPK active), saving energy. By controlling upstream kinases, mTORC1 indirectly controls AMPK. The relationship with HIF-1α is also complicated: mTORC1 activation can increase the translation of HIF-1α in normoxic conditions, especially in cells that are rapidly growing or inflammation-activated, but hypoxia stabilizes HIF-1α (Schneider et al. 2008). Other metabolic sensors and transcription factors are also deeply ingrained, in addition to these three. These metabolic processes frequently interact with the hallmarks of inflammatory signaling, NF-κB, and signal transducers and activators of transcription. For instance, activation of NF-κB can trigger glycolytic enzymes, and metabolites from glycolysis may provide feedback to regulate NF-κB activity (Jung et al. 2025). Nuclear receptors called PPARs (Peroxisome Proliferator-Activated Receptors), especially PPAR-γ, cause FAO and OXPHOS and are frequently linked to anti-inflammatory traits, indicating a counter-regulatory role against the glycolytic shift (Chawla 2010). The NAD+/NADH ratio and sirtuins, which are NAD+-dependent deacetylases, are also important metabolic checkpoints that influence the activity of AMPK, HIF-1α, and other metabolic enzymes, connecting cellular redox state to immunological responses and metabolic programming (Houtkooper et al. 2012).

The capacity of these regulators to impose certain functional phenotypes highlights their substantial relevance to immune cell metabolism, especially in microglia. Such metabolic toggles also play a major role in the regulation of the transition between homeostatic and activated microglia phenotypes in neuroinflammation (Muzio et al. 2021). Pro-inflammatory (M1-like) microglia, which are commonly seen in cases of acute injury or chronic neurodegenerative illness, have a metabolic profile that includes a coordinated decrease in OXPHOS and enhanced aerobic glycolysis, which is fueled by activated mTORC1 and HIF-1α (Xu et al. 2023). The quick ATP needed for pro-inflammatory cytokine production and release (e.g., IL-1β, TNF-α) and reactive oxygen/nitrogen species, both of which cause neurotoxicity and enhanced neuronal damage, is produced by this glycolytic dependence.(Amruta et al. 2020). On the other hand, pro-resolving/anti-inflammatory (M2-like) microglia, which are essential for tissue repair, debris removal, and neurotrophic supply, generally have higher levels of OXPHOS and fatty acid oxidation, which are linked to enhanced AMPK activity and suppressed mTORC1 signaling (Jha et al. 2015). Constant energy demands and the synthesis of growth factors and anti-inflammatory mediators (such as TGF-β and IL-10) are supported by this metabolic phenotype (Al-Kuraishy et al. 2023). These pathways have been shown to rewire microglial phenotypes both in vitro and in vivo, and they can be blocked or activated in experiments using pathway-specific inhibitors or stimulators. Inhibiting glycolysis or HIF-1α, for instance, has been shown to decrease pro-inflammatory microglial activation and enhance results in AD and PD models (Alrouji et al. 2024a). In contrast, microglia can be reoriented towards a more neuroprotective, healthy phenotype through AMPK activation or fatty acid oxidation stimulation (Al-Kuraishy et al. 2025). The implementation of targeted therapeutic interventions for modifying microglial function in an effort to reduce neuroinflammation and slow the pathogenesis of neurodegenerative diseases is made possible by an understanding of how such transitions are regulated by downstream metabolic regulators (Al-Kuraishy et al. 2025).

The complex crosstalk between cellular metabolites and metabolic sensors is crucial in determining microglial phenotype. The metabolites are not only substrates but immunometabolites that directly shape central inflammatory pathways like HIF-1α, NLRP3, NF-κB, and sirtuins. The paradigmatic case in point is the TCA cycle intermediate succinate. In pro-inflammatory (M1-like) microglia, a truncated TCA cycle or reversed electron flux leads to succinate buildup (Li et al. 2024a). This metabolite acts as a signal through competitive inhibition of Prolyl Hydroxylase Domain (PHD) enzymes, otherwise tagging HIF-1α for proteasomal degradation. This induced stabilization of HIF-1α preserves the glycolytic program and induction of pro-inflammatory effectors, such as the major cytokine IL-1β. Furthermore, succinate has been recognized as a key endogenous ligand mediating assembly and activation of the NLRP3 inflammasome, important for IL-1β maturation and secretion, firmly connecting succinate with acute neuroinflammation (Li et al. 2024a). Conversely, redox state and biosynthetic building blocks regulate anti-inflammatory activities. The intracellular ratio of NAD+/NADH reflects sufficiency in energy, virtually governing activity in Sirtuins (NAD+-dependent deacetylases). A high NAD + status is required to deacetylate the p65 subunit of NF-κB by SIRT1 and SIRT2, thereby inhibiting the master inflammatory pathway and evoking a pro-resolving phenotype (Cantó et al. 2015).

Moreover, glycolytic end-product lactate is not waste but an important signaling molecule. Recent findings suggest that lactate can elicit neuroprotective responses via the activation of HIF-1α, which surprisingly suppresses the NF-κB pathway in cerebral ischemia. Lactate also enhances lactylation, a histone epigenetic modification that regulates microglial transcriptional states (Lee 2021). Finally, TCA cycle intermediate citrate, diverted to the cytoplasm, is cleaved to yield acetyl-CoA, providing the substrate needed for fatty acid and lipid synthesis (mTOR-dependent) and serving as the primary source of acetyl groups for Histone Acetyltransferases, which regulate the epigenetic state for chronic inflammatory gene expression (Williams and O’Neill 2018).

The integration of the AMPK/PGC-1α/SIRT3 cascade provides an elegant mechanistic framework that bridges cellular energy sensing to long-term mitochondrial well-being and anti-inflammatory microglial activity (Abu Shelbayeh et al. 2023). As the cell’s master energy sensor, activated AMPK responds to a reduced ATP: AMP ratio by initiating a general pro-survival program. Key to the program is the phosphorylation and activation of PGC-1α. PGC-1α is a master transcriptional co-regulator, stimulating nuclear and mitochondrial gene expression in mitochondrial biogenesis and enhancing OXPHOS capacity, characteristic of the neuroprotective, pro-resolving (M2-like) microglial phenotype (Abu Shelbayeh et al. 2023; Mishra et al. 2025).

Notably, PGC-1α is also a major inducer of mitochondrial sirtuin, SIRT3. Being an NAD+-dependent deacetylase, SIRT3 is found within the mitochondria where it ensures maximal activity by activating and deacetylating essential TCA cycle and OXPHOS enzymes to improve efficiency and quality of mitochondrial metabolism (Gao and Shen 2023). This cooperative activation, AMPK signals energy stress, PGC-1α produces new mitochondria, and SIRT3 maintains them properly working, is crucial for adapting to the high, prolonged energy demands of phagocytosis and production of anti-inflammatory mediators (e.g., TGF-β, IL-10), and constitutes a potent counter-regulatory response to the glycolytic shift of neurotoxic activation (Xu et al. 2024b).

## Microglial metabolic phenotypes in health and disease

Microglia have a highly malleable metabolism in the dynamic CNS milieu, assuming several metabolic phenotypes that greatly influence their functional identity in both health and illness. Whether microglia promote tissue repair and have neuroprotective effects or lead to cytotoxic neuroinflammation and neurodegeneration depends critically on this metabolic reprogramming. Though increasingly acknowledged as an oversimplification of a continuum, one of the first theoretical models commonly used to characterize these functional states makes a distinction between “pro-inflammatory” (M1-like) and “anti-inflammatory/resolving” (M2-like) microglial phenotypes, each of which is sustained by distinct metabolic signatures (Amruta et al. 2020).

The pro-inflammatory (M1-like) phenotype of microglia is characterized by a rapid metabolic shift towards aerobic glycolysis in response to acute pathological stimuli, such as danger-associated molecular patterns (DAMPs) from damaged cells (e.g., ATP, HMGB1), pathogen-associated molecular patterns (PAMPs) of invasive microbes (e.g., lipopolysaccharide, LPS), or misfolded proteins typical of neurodegenerative diseases (e.g., Aβ in AD, alpha-synuclein in PD) (Sun et al. 2023). Even when there is enough oxygen present to permit OXPHOS, in analogy with cancer cells, it involves enhanced glucose absorption with a preference for lactate metabolism (Jung et al. 2025). This metabolic change is an ideal adaptation that permits rapid and powerful production of pro-inflammatory mediators rather than a pointless use of energy. ATP is instantly available to power energy-demanding processes such as the transcription and translation of pro-inflammatory cytokines (e.g., TNF-α, IL-1β, IL-6) and chemokines due to the rapid glycolytic flux (Muzio et al. 2021). During normoxic inflammatory conditions, transcription factors such as HIF-1α frequently cause the overexpression of key enzymes of the glycolytic pathway, including hexokinase (HK), phosphofructokinase (PFK), and lactate dehydrogenase (LDH) (Qannita et al. 2024). Some glycolytic intermediates have a direct impact on inflammatory signaling in addition to providing ATP. For instance, succinate is a signaling molecule and an intermediary of the TCA cycle that can build up due to reverse glycolytic flow. Its buildup increases the cascade of inflammation by stabilizing HIF-1α and activating the NLRP3 inflammasome, which in turn causes proteolytic cleavage and the release of mature IL-1β and IL-18 (Anderson et al. 2023). Furthermore, dependence on glycolysis can increase the production of ROS through a variety of mechanisms, such as changed mitochondrial dynamics and stimulation of NADPH oxidase, which can be harmful to neurons in and of themselves and contribute to the “oxidative burst” feature of M1-like microglia (Alrouji et al. 2024b). Therefore, this metabolic state maintains a strong, quick inflammatory response that aims to eliminate impending danger, but if it persists or is dysregulated, it becomes the main cause of chronic neuroinflammation and spreads neurodegenerative mechanisms by maintaining neuronal death and disrupting synaptic function (Muzio et al. 2021).

However, microglia adopt an anti-inflammatory/resolving (M2-like) phenotype, which is defined by a metabolic shift towards OXPHOS and FAO, to sustain tissue repair, debris clearance, and inflammation resolution activities (Mehla and Singh 2019). This metabolic state, which denotes a change towards healing and homeostatic activity, is typically brought on by growth factors, necrotic cells, or anti-inflammatory cytokines like IL-4, IL-13, or IL-10. M2-like microglia demonstrate increased mitochondrial respiration, which is predicated on the effective breakdown of fatty acids and glucose via the electron transport chain and TCA cycle to create a greater, longer-lasting ATP supply (Jha et al. 2015). Because it offers a very effective and durable source of energy, fatty acid oxidation in particular is essential for powering these long-term processes. Transcriptional regulators such as peroxisome proliferator-activated receptor gamma (PPAR-γ) and PGC-1alpha, which support mitochondrial biogenesis and metabolism, often regulate the induced FAO and OXPHOS enzymes (e.g., CPT1) (Al-Kuraishy et al. 2023). In terms of metabolism, this phenotype is linked to a more robust and healthy TCA cycle, which is the basis for the synthesis of amino acids and other building blocks required for extracellular matrix remodeling and tissue healing (Jung et al. 2025). M2-like microglia have phagocytic functions that reduce inflammation and provide a healthy microenvironment by removing apoptotic cells, misfolded proteins (including Aβ and alpha-synuclein), and cellular detritus (Miao et al. 2023a). Additionally, they release neurotrophic factors (BDNF, IGF-1) and anti-inflammatory cytokines (IL-10, TGF-β) that promote synaptogenesis, neuronal survival, and overall CNS repair and plasticity (Al-Kuraishy et al. 2024c). To stop neuroinflammation and avoid the transition to persistent, pathogenic immunological responses that are typical of most neurodegenerative disorders, microglia must be able to flip to this M2-like, OXPHOS/FAO-resume metabolic state. One of the main causes of chronic neuroinflammation and the advancement of disease is the dysregulation of such metabolic flexibility, where microglia are unable to efficiently transition to a pro-resolving phenotype. This highlights the therapeutic potential of treatment plans intended to restore such metabolic balance (Al-Kuraishy et al. 2024b). Furthermore, recent studies highlight that oligodendrocytes are not merely passive targets of injury but actively participate in regulating the neuroinflammatory environment, often through metabolic crosstalk with microglia (Zou et al. 2023; Sasmita et al. 2024; Wu et al. 2025; Huang et al. 2025).

It is important to remember that microglial activation is a very dynamic process, even if the M1-like (pro-inflammatory) and M2-like (anti-inflammatory/resolving) phenotypes are utilized as conceptual anchors in this discussion, especially in relation to their distinct metabolic profiles. The M1-like and M2-like classifications should be viewed as the extremes of this functional and metabolic continuum rather than as permanent, definitive states. Microglial phenotypes exist along a complicated range of activation states.

While M1-like/M2-like remains a useful conceptual framework, recent advances in immunometabolism emphasize that these phenotypic states are tightly controlled by a very plastic metabolic continuum governed by central energy-sensing pathways (Strizova et al. 2023). Commitment of microglia to the pro-inflammatory (M1-like) program is redundantly orchestrated by the engagement of the mTORC1, a master rheostat that ties nutrient availability to cellular growth and biosynthetic demands. When stimulated by DAMPs or misfolded proteins, mTORC1 activation coordinates with HIF-1α to induce the expression of glycolytic enzymes, guaranteeing the rapid flux of glucose necessary to support the rapid synthesis and secretion of pro-inflammatory cytokines at the expense of mitochondrial efficiency (Strizova et al. 2023). This acute anabolic switch also bridges glycolysis intermediates and inflammatory signaling, such as the stabilization of HIF-1α and activation of the detrimental NLRP3 inflammasome cascade by succinate accumulation. In stark contrast, phenotypic switching to the anti-inflammatory/resolving (M2-like) phenotype is absolutely dependent upon the reciprocal activation of AMPK. As the cell’s master energy sensor, AMPK is activated by either energy stress or pharmaceutical activators and increases catabolic processes to restore energy homeostasis (Strizova et al. 2023). At the mechanistic level, AMPK simultaneously inhibits the pro-inflammatory mTORC1 pathway and preserves the energy-sparing OXPHOS program, in large part by conducting Fatty Acid Oxidation (FAO) through upregulation of enzymes including CPT1. This adaptive metabolic mode provides the long-term ATP provision and anabolic precursors required for long-duration events, including intense phagocytic removal of disease-associated aggregates (Aβ, alpha-synuclein), extracellular matrix remodeling, and neurotrophic factor secretion (Garcia and Shaw 2017). The inability of microglia to undergo this necessary metabolic shift from mTORC1-driven glycolysis to AMPK-driven OXPHOS/FAO, a failure referred to as metabolic rigidity, is now recognized as a driving force behind chronic neuroinflammation and disease development, positioning these central metabolic sensors at the forefront of therapeutic targets (Garcia and Shaw 2017).

## Disease-specific microglial reprogramming in neurodegeneration

Research on the complex connection between microglial metabolic remodeling and the etiology of neurodegenerative illness is quickly growing. It describes how these resident immune cells alter their energy landscapes in order to either exacerbate or lessen pathology. Although neuroinflammatory indicators are present in all neurodegenerative diseases, each one has unique metabolic needs and microglial expression patterns.

### Alzheimer’s disease

**Fig. 2:**
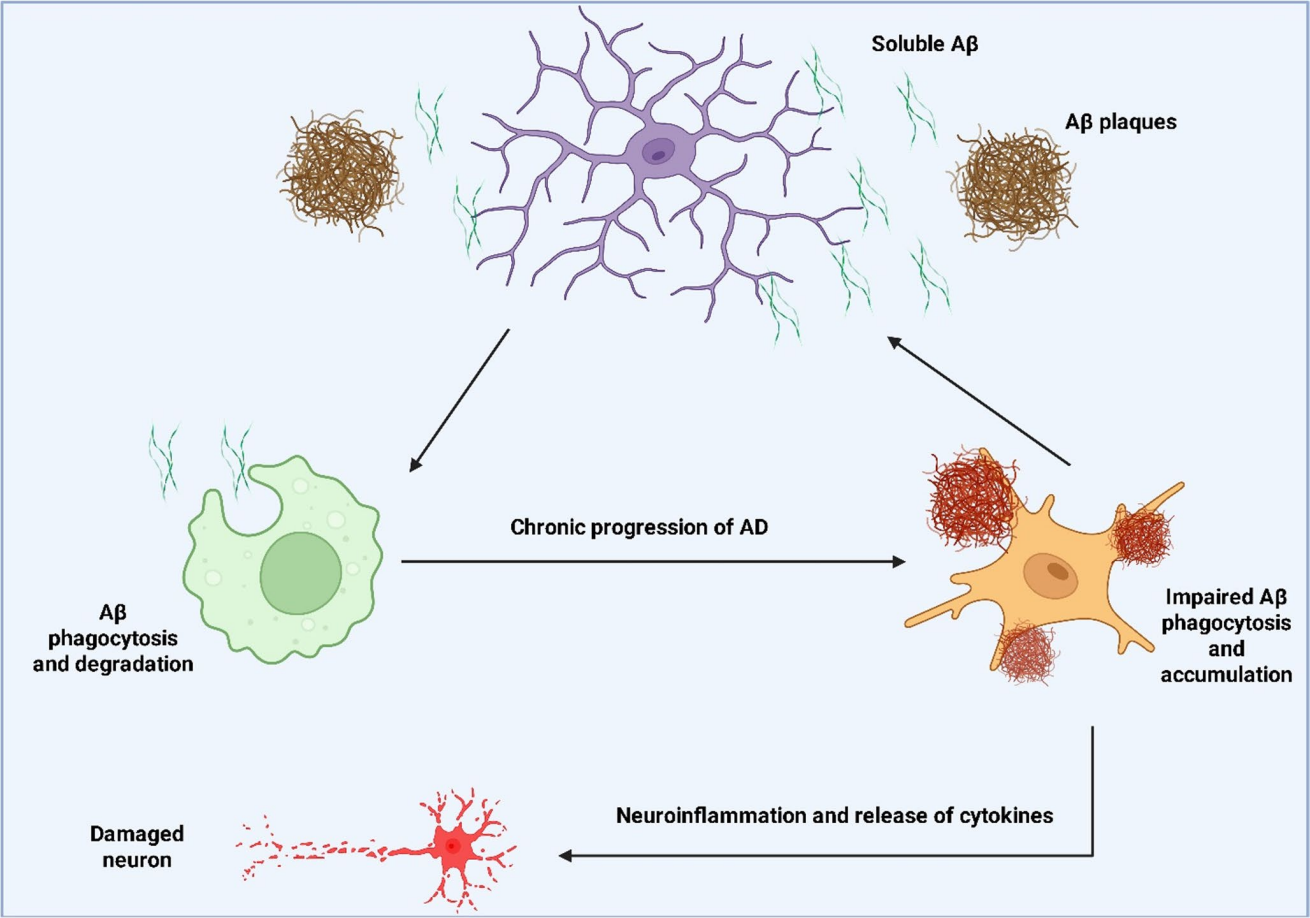
The dual role of microglia in Alzheimer’s Disease. Initially, microglia attempt to clear Aβ plaques. However, chronic exposure leads to impaired phagocytosis, Aβ accumulation, and neuroinflammation, contributing to neuronal damage and disease progression

Microglia play a crucial but contradictory function in this deadly neurodegenerative disease, which is characterized by intracellular neurofibrillary tangles made of hyperphosphorylated tau protein and extracellular Aβ plaques (Zhang et al. 2024) [Figure 2]**.** First attracted to Aβ plaques, microglia try to phagocytose these aggregates and usually settle into a condition known as disease-associated microglia or microglial neurodegenerative phenotype. On the other hand, prolonged exposure to tau tangle and Aβ causes particular and harmful metabolic alterations in microglia, which result in dysregulated clearance pathways and protracted pro-inflammatory conditions. Studies have demonstrated that, even when faced with high energy demands to cope with pathological loads, AD microglia usually tolerate poor glucose metabolism, decreased glucose uptake, and decreased OXPHOS function (Xu et al. 2023). Mitochondrial dysfunction, which is typified by abnormal mitochondrial dynamics (i.e., hyperfission), decreased ATP synthesis, and elevated ROS formation, frequently coexists with this metabolic dysfunction (Al-Kuraishy et al. 2023a). Microglial mitochondria are directly affected by the accumulation of Aβ oligomers and fibrils, which depolarize them and impair their respiration, further depleting them of their energy-demanding functions such as phagocytosis (D’Alessandro et al. 2025). Because abnormal mitochondria may generate DAMPs (such as mitochondrial DNA) that activate the NLRP3 inflammasome and prolong the inflammatory response, this metabolic dysfunction is the driving force behind chronic inflammation (Anderson et al. 2023). Additionally, AD microglia develop lipid droplets and have aberrant lipid metabolism (Li et al. 2025). These lipid droplets are linked to an inadequate phagocytic condition that is involved in the poor clearance of tau and Aβ, and they can also be a source of inflammatory lipids (Li et al. 2025). In AD microglia, the transcription factor PPAR-γ, which is typically advantageous for lipid catabolism and an anti-inflammatory phenotype, is frequently dysregulated, which exacerbates lipid buildup and the inflammatory response (Li et al. 2025). Because of metabolic pathology, this chronic inflammatory state leads to a vicious cycle in which microglia, rather than removing pathology, become a site of neurotoxicity, generating pro-inflammatory cytokines (TNF-α, IL-1β) and further contributing to synaptic loss and neuronal damage, ultimately speeding up the progression of AD disease (Valiukas et al. 2025). Thus, therapeutic approaches that promote lipid breakdown or restore microglial glucose and mitochondrial metabolism are becoming more and more viable options for improving Aβ/tau clearance and preventing neuroinflammation in AD.

In AD, microglial pathology is very stage-dependent, indicating a transition over time from protective activity to long-term neurotoxicity. Early in the amyloid deposition process, microglia actively attempt to remove Aβ and mitigate damage by adopting a temporary M2-like/phagocytic profile, commonly referred to as disease-associated microglia (Miao et al. 2023b). However, this beneficial metabolism breaks down as the disease worsens and the load of hyperphosphorylated tau and Aβ builds up. Despite high energy demands, the prolonged pathogenic load triggers a crucial temporal shift towards a dysfunctional M1-like phenotype, which defective OXPHOS and poor glucose metabolism typify. Severe mitochondrial hyperfission and the buildup of lipid droplets associated with a breakdown in phagocytic clearance are characteristics of this latter, chronic stage (Miao et al. 2023b). The main cause of the non-resolving neuroinflammation in late-stage AD is this metabolic collapse, which turns microglia from efficient scavengers into a source of neurotoxicity that hastens synapse loss and neuronal destruction. To stop this harmful phenotypic shift, treatment approaches must concentrate on re-establishing metabolic integrity (Miao et al. 2023b).

Failures in particular metabolic pathways are the molecular cause of the transition from protective to neurotoxic microglia. There is evidence that the *APOE4* genotype contributes to the development of AD by causing a significant build-up of toxic lipid droplets in microglia, which damages the phagocytic machinery that depends on OXPHOS and encourages chronic inflammation (Chen et al. 2025). We also discuss recent research demonstrating that the production of neurotoxic lipids is directly caused by the disruption of the integrated stress response caused by tau and Aβ. By presenting AD pathogenesis as a direct result of microglial immunometabolic collapse, which is fueled by abnormal lipid and mitochondrial dynamics, these contributions bolster our case and identify these pathways as important targets for treatment (Flury et al. 2025).

In AD, microglial metabolic disease is centered on lipid metabolism axes, lysosomal degradation, and mitochondrial quality control, collectively driving failure of Aβ and Tau clearance. The core disease-associated microglia state is in most instances synonymous with lipid droplet (LD) Accumulation (Jung et al. 2025). This occurs when the phagocytic load of Aβ-associated lipids (e.g., cholesterol esters) exceeds the microglial capacity of fatty acid oxidation (FAO), on a regular basis due to defective mitochondria. The ensuing LD load is a major metabolic stressor, compelling the microglial state into perpetual dysfunction. Compounding this, AD pathology disrupts lysosome/autophagy flux; Aβ uptake often leads to lysosomal membrane permeabilization, compounded by genetic risk factors such as deficiency in TREM2, which normally guarantees efficient lysosomal catabolism (Jung et al. 2025). This combined phagocytic and lysosomal breakdown codes the warranty of efficient Aβ clearance. The sequestered toxic burden and the LD-induced stress overwhelm the Mitochondrial Quality Control machinery, leading to Mitophagy failure and accumulation of defective, ROS-permeable mitochondria. These defective organelles are potent generators of reactive oxygen species (ROS), which are the critical upstream signal to induce the pro-inflammatory NLRP3 inflammasome, thus initiating a vicious cycle of metabolic failure inducing inflammation followed by phagocytic paralysis (Jung et al. 2025).

### Parkinson’s disease

Microglia are key players in the pathophysiology and development of this individual or progressive neurodegenerative disease, which is primarily characterized by the degeneration of dopaminergic neurons in the substantia nigra pars compacta and the sequestration of misfolded α-synuclein into Lewy bodies (Alrouji et al. 2024a; Alshahrani et al. 2025a). To remove misfolded protein and cellular detritus, microglia are first drawn to areas of neuronal injury and α-synuclein disease (Ma et al. 2019; Alshahrani et al. 2025b). However, extended exposure to aggregated α-synuclein, especially its oligomers, can cause microglia to become persistently activated, which alters their surveillance capabilities and pushes them into a pro-inflammatory profile (Roodveldt et al. 2024). This sustained activation is strongly associated with certain metabolic changes in microglia. According to studies, α-synuclein aggregates can cause a glycolytic shift in microglia, resembling the M1-like phenotype, such that the cells can quickly obtain ATP for the production of pro-inflammatory cytokines and ROS (Xu et al. 2023; Al-Kuraishy et al. 2024b). Usually, this metabolic reprogramming is accompanied by mitochondrial dysfunction, such as decreased mitochondrial membrane potential, impaired OXPHOS, and elevated oxidative stress, which also impairs microglial health and function (Al-Kuraishy et al. 2025). Lysosomal failure and aberrant mitophagy (autophagic selective destruction of defective mitochondria) are key components of Aβ pathogenesis (Xu et al. 2023). For microglia to break down ingested α-synuclein, lysosomal activity must be intact. However, aggregated α-synuclein itself can directly impair lysosomal function and cause a dysregulated build-up of defective mitochondria, which promotes the duration of inflammation and decreases the ability of microglia to remove illness proteins (Ali et al. 2023). The metabolic fallout from this mitochondrial and lysosomal dysfunction leads to a chronic pro-inflammatory state, where microglia release neurotoxic chemicals that promote dopaminergic neuron degeneration instead of having the capacity to remove α-synuclein (Alrouji et al. 2024a). As an example, when the NLRP3 inflammasome in microglia is activated, usually due to lysosomal degradation or malfunctioning mitochondria, IL-1β, a strong pro-inflammatory cytokine implicated in Aβneurodegeneration, is released (Alrouji et al. 2023; Chen et al. 2023). Therefore, in order to combat α-synuclein pathology and protect dopaminergic neurons in PD, treatment for the restoration of microglial lysosomal function, induction of mitophagy, or modification of their metabolic processes to create an anti-inflammatory, pro-clearance phenotype is essential (Alrouji et al. 2023; Chen et al. 2023) [Table 1].

**Table 1:** A table demonstrates the different roles of microglia in parkinson’s disease

Study	Model/study	Key Findings	Metabolic findings	Functional outcome	Reference
**Microglial activation in PD brains**	Human PD patients	Post-mortem studies show activated microglia in the substantia nigra of PD patients	Activated state implies a shift to a dysfunctional M1-like metabolism	Activated microglia in the substantia nigra	(McGeer et al. 1988)
***PINK1/Parkin*** **mutations impair microglial mitophagy**	PD Gene Mutation Models (in vitro/in vivo)	Loss of *PINK1* or *Parkin* disrupts mitochondrial quality control in microglia, exacerbating neuroinflammation.	Loss of mitochondrial quality control (Impaired Mitophagy)	Exacerbated neuroinflammation	(Matheoud et al. 2019)
***LRRK2*** **mutations alter microglial function**	LRRK2-G2019S mutation models	*LRRK2-G2019S* mutation increases microglial cytokine release and promotes neurotoxicity in PD models.	Increased cytokine release suggests a shift towards aerobic glycolysis (M1-like)	Increases microglial cytokine release and promotes neurotoxicity	(Moehle et al. 2012)
**Microglial Activation & Neuroinflammation**	General PD context	PD is characterized by activated microglia and increased levels of proinflammatory factors.	Shift to M1-like metabolism (Glycolysis)	Increased levels of proinflammatory factors	(Xu et al. 2024a)
**Microglial Phagocytosis of α-Synuclein**	General PD context	Microglia play a critical role in clearing alpha-synuclein aggregates, the hallmark pathology of PD.	Phagocytosis requires high energy, suggesting a need for efficient OXPHOS/FAO	Critical role in clearing alpha-synuclein aggregates	(Ho 2025)
**Microglia and Peripheral Immune System Interaction**	Systemic/Gut-Brain Axis Interaction	Interactions with immune cells that infiltrate the body (such as T cells and B cells) and the substances they emit can also affect microglial activation and add to the neuroinflammatory milieu in PD.	Activation affected by peripheral immune substances (e.g., T cells)	Adds to the neuroinflammatory milieu in PD	(Ciolac and Gonzalez-Escamilla 2025)

Critical stage-dependent and temporal dynamics are hallmarks of microglial dysfunction in PD. Microglia try to phagocytose misfolded α-synuclein in early PD, demonstrating a comparatively neuroprotective role. A temporal transition towards a chronic, dysfunctional state is driven by the extended existence of α-synuclein oligomers (Lind-Holm Mogensen et al. 2025). Along with severe lysosomal and mitochondrial dysfunction, this shift is characterized by a non-resolving glycolytic switch (M1-like metabolism) to enhance the synthesis of pro-inflammatory cytokines. The accumulation of damaged mitochondria results from this breakdown of α-synuclein, which hinders mitophagy and feeds the NLRP3 inflammasome’s persistent activity. Microglia lose their ability to be cleared in this late-stage pro-inflammatory profile, turning into a source of neurotoxicity that actively promotes dopaminergic neuron degeneration (Lind-Holm Mogensen et al. 2025). Therefore, a key component of therapeutic intervention is stopping this metabolic and lysosomal breakdown.

In PD, microglial dysfunction is intrinsically linked to profound defects in cell quality control and directly participates in α-Synuclein (α-Syn) clearance, and in chronic neuroinflammation. This mechanism consistently initiates with mitochondrial quality control (Mitophagy) impaired, a defect highlighted by PD-linked mutations in genes such as *PINK1 and Parkin* (Arena et al. 2022). Mitophagy-deficient microglia contain accumulations of dysfunctional mitochondria, which incapacitate the OXPHOS-mediated energy metabolism involved in homeostatic functions and enables a low-grade inflammatory state. This is compounded by profound defects in the lysosome/autophagy pathway, particularly due to defective GBA1 (Glucosylceramidase) function, the most common genetic risk factor in PD. Defective GBA1 impairs the breakdown of α-Syn effectively, leading to its accumulation and transfer within the CNS (Arena et al. 2022). The aggregated and misfolded α-Syn, together with the cellular stress and disrupted FAO, then promotes lipid droplet (LD) formation, marking a defective LDAM state. This vicious cycle culminates in the activation of the NLRP3 inflammasome, which is uniquely triggered by the accumulated α-Syn fibrils and the surplus ROS produced by the malfunctioning mitochondria. Consequently, in PD, defective quality control mechanisms (mitophagy and lysosome) drive metabolic degradation (LD accumulation) which ultimately degrades microglial phagocytic activity and sparks neurotoxic inflammation (Arena et al. 2022).

### Multiple sclerosis

**Fig. 3:**
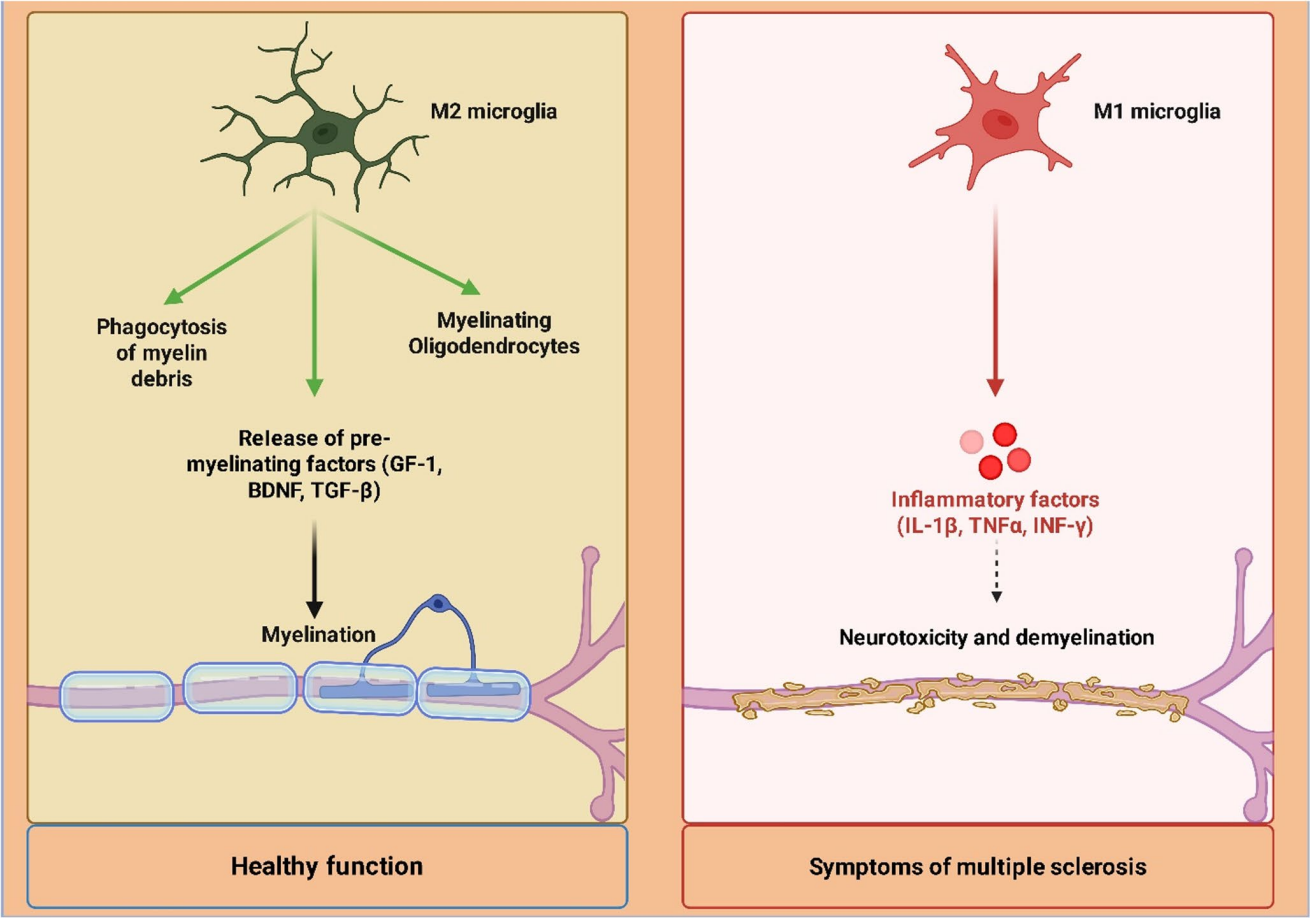
The contrasting roles of microglia in healthy function versus MS. On the left, M2 microglia exhibit a reparative phenotype, promoting healthy myelination by phagocytosing myelin debris, supporting myelinating oligodendrocytes, and releasing pro-myelinating factors like GF-1, BDNF, and TGF-β. This contributes to healthy myelin. On the right, M1 microglia adopt a pro-inflammatory state, releasing inflammatory factors (IL-1β, TNF-α, INF-γ) that lead to neurotoxicity and demyelination, characteristic of MS symptoms. The figure highlights the critical balance of microglial states in neurological health and disease

Multiple sclerosis is a neurological dysfunction that results from immune-mediated damage to myelin and axons in an inflammatory demyelinating disease of the CNS (Dutta and Trapp 2011). The hallmark of MS pathophysiology is the infiltration of peripheral macrophages and microglia, with dynamic changes in their metabolic status as the disease progresses through neurodegenerative (progressive) and inflammatory (relapsing) phases (Dutta and Trapp 2011; Abulaban et al. 2025a). Microglia and macrophages of MS lesions mostly exhibit a pro-inflammatory (M1-like) metabolic profile during the acute inflammatory phase, with a tendency towards aerobic glycolysis (Muzio et al. 2021). Such a glycolytic burst promotes the rapid generation of chemokines, reactive oxygen/nitrogen species, and pro-inflammatory cytokines (such as TNF-α and IFN-γ), which result in oligodendrocyte destruction, demyelination, and axonal injury (Amruta et al. 2020). In such inflammatory cells, succinate accumulation and HIF-1α induction are also commonly observed (Jha et al. 2015). On the other hand, microglia and macrophages may polarize towards an anti-inflammatory/resolving (M2-like) phenotype in remission or attempted remyelination conditions, with a greater dependence on OXPHOS and FAO (Mehla and Singh 2019; Al-Kuraishy et al. 2023). Energy-demanding processes such as myelin debris clearance, phagocytosis of cellular waste, and the production of pro-remyelinating substances (e.g., IGF-1, BDNF, TGF-β) that support oligodendrocyte differentiation and myelin repair are supported by this metabolic profile (Xu et al. 2023) [Figure 3]**.** However, in progressive MS, the cells usually either fail to correctly shift to a constant pro-resolving metabolic state or experience inappropriate chronic activation, which results in low-grade inflammation and poor remyelination (Xu et al. 2023). Myelin debris buildup is also physiologically challenging to eliminate, leading to microglial dysfunction and a chronic inflammatory milieu (Xu et al. 2023).

In MS, microglial function is phase-dependent and influenced by crucial temporal dynamics. The primary macrophage and microglial phenotype during the acute, recurrent inflammatory phase is M1-like, which is fueled by an aggressive shift to aerobic glycolysis to promote the quick synthesis of pro-inflammatory chemicals (O’Loughlin et al. 2018). In contrast, a brief transition to the M2-like phenotype takes place during attempted repair in order to improve the removal of myelin debris and the release of pro-remyelinating proteins. The main issue is that this pro-resolving state is not maintained. Low-grade inflammation, and eventually remyelination failure are the results of a metabolic state trapped between M1- and M2-like profiles caused by the persistence of challenging debris and improper chronic activation (O’Loughlin et al. 2018). For therapeutic benefit, the metabolic switch must be targeted in order to maintain the pro-resolving M2-like phenotype.

The failure of the M2-like shift in progressive MS has been addressed in recent research. It was demonstrated that the microglial endolysosomal and metabolic capacity is overwhelmed by the persistent phagocytosis of myelin debris in progressive lesions (Van Der Vliet et al. 2024). Failure of remyelination and accelerated neurodegeneration are directly linked to the abnormal buildup of lipid droplets and cholesterol esters, which results in the “foamy microglia” phenotype. In order to modify the metabolic switch and support the neuroprotective, pro-resolving phenotype, mitochondrial complex I activity and reverse electron transport, which propel persistent pro-inflammatory activation in progressive MS microglia (Van Der Vliet et al. 2024).

### Ischemic stroke and traumatic brain injury

Both short-term and long-term rates are determined by the quick and profound metabolic reprogramming of microglia (Sun et al. 2023). Microglia are among the first immune cells to activate in the minutes to hours following a stroke, which is an abrupt stoppage of blood flow to a portion of the brain, or TBI, which is brought on by mechanical stress (Sun et al. 2023). Acute inflammation and secondary brain injury can result from this first response, which is frequently characterized by a glycolytic burst that produces fast ATP to support immediate pro-inflammatory processes such as the production of cytokines, chemokines, and ROS (Xu et al. 2023). Their quick migration into the injury site and early phagocytosis of cellular debris depend on this metabolic change. Nonetheless, the microglia’s subsequent metabolic pathway plays a critical role in weighing the likelihood of recovery against the extent of damage. Microglia can transition to an even more reparative, OXPHOS-dependent state in the penumbra zone after ischemic stroke, where tissue is reversible, removing waste and encouraging angiogenesis and neurogenesis (Muzio et al. 2021). But in the days and weeks after the initial damage, chronic inflammation, spreading excitotoxicity, BBB rupture, and neuronal death might be triggered by persistent or excessively glycolytic metabolism (Kim et al. 2015). Long-term neurological impairments and an increased risk of neurodegeneration can result from microglia being driven to a chronic pro-inflammatory metabolic state by repeated exposure to DAMPs and the diverse inflammatory milieu associated with TBI (Kim et al. 2015). Microglia’s phagocytic activity, secretory capacity to release neurotrophic factors, and tissue remodeling function are all directly impacted by their metabolic state after injury (Al-Kuraishy et al. 2024c). To reduce secondary injury and improve long-term functional recovery following stroke and TBI, therapeutic interventions that target the modulation of microglia’s acute metabolic response, thereby enabling a prompt shift from pro-inflammatory glycolysis to pro-resolving OXPHOS/FAO, are being investigated.

Acute CNS damage triggers a very time-sensitive and stage-dependent microglial response. To support acute inflammatory responses, microglia quickly transition to a highly glycolytic (M1-like) state in the first few hours after damage (Kawabori and Yenari 2014). To assist angiogenesis and waste elimination, there is a rapid metabolic shift over time towards a reparative phenotype that is M2-like and dependent on OXPHOS. A prolonged chronic pro-inflammatory glycolysis occurs when this switch is not completed, especially at the core injury site or after persistent DAMPs. This is the main distinction, which eventually results in poor functional recovery, subsequent damage spread, and long-term neurotoxicity (Kim and Lee 2025). The outcome is therefore determined by the metabolic trajectory: chronic damage is caused by a protracted glycolytic state, whereas recovery is facilitated by a quick shift to OXPHOS-driven repair.

## Gut-microbiome-derived metabolites and microglial metabolic reprogramming

Short-chain fatty acids (SCFAs), such as butyrate, propionate, and acetate, which are byproducts of bacterial fermentation of dietary fiber, are the main mediators of the crucial connection between gut-microbiome-derived metabolites and microglial metabolic reprogramming (Głowacka et al. 2024). These lipophilic metabolites can penetrate the BBB and directly affect microglia, changing their functional status. By enhancing the energy profile of the cells, SCFAs, butyrate in particular, mechanistically support the pro-resolving M2-like phenotype. By acting as a histone deacetylase inhibitor, butyrate strengthens the metabolic bias of the M2-like state by causing epigenetic modifications that increase the expression of genes involved in fatty Acid OXPHOS (Głowacka et al. 2024). The persistent, pro-inflammatory glycolytic (M1-like) state observed in chronic neurodegenerative disorders, on the other hand, can be attributed to dysbiosis that results in a decrease in SCFA production. This provides a compelling therapeutic avenue to target microglial dysfunction through nutritional and microbiome interventions.

To elaborate on the process, these SCFAs influence microglial cells directly through G-protein coupled receptors (GPCRs), specifically GPR43, in addition to epigenetic regulation. GPR43 activation sets off signaling cascades that regulate the transition from pro-inflammatory glycolysis to pro-resolving oxidative metabolism by balancing the expression of metabolic enzymes (Głowacka et al. 2024). Additionally, research indicates that C-acetate can cross the blood-brain barrier and reach the microglial TCA cycle, where it directly supplies intermediates to power OXPHOS [76]. A healthy gut-derived metabolite profile is directly linked to neuroprotection because this restoration of mitochondrial function is essential for supplying the sustained energy required for the energy-demanding processes of the M2-like phenotype, including as phagocytosis and tissue repair factor release (Erny et al. 2021).

## Therapeutic strategies

A shift from general anti-inflammatory strategies to more focused interventions targeted at restoring microglial homeostasis and enhancing protective activities has been made possible by the recent discovery of the metabolic reprogramming of microglia in neurodegenerative disease. The capacity of a wide variety of pharmacological modulators to specifically alter microglial metabolic activity and, consequently, their functional phenotypes is being studied.

### AMPK activators

**Fig. 4:**
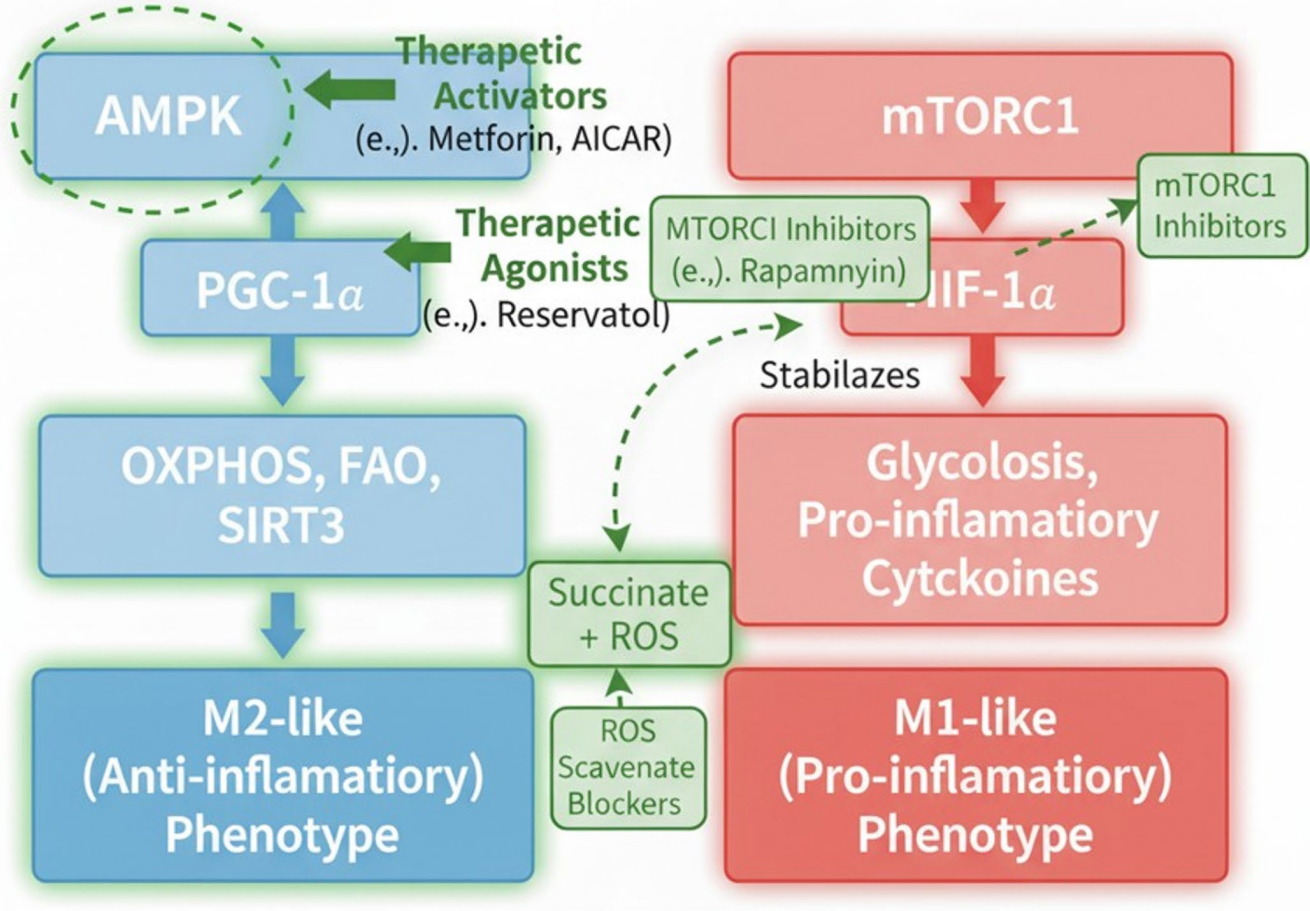
Integrated Therapeutic Approaches in Microglial Metabolic Control. This figure depicts major sites of therapeutic intervention in the central metabolic control network of microglia, designating attempts to shift their functional phenotype. The blue pathway underscores mechanisms favoring an M2-like (anti-inflammatory) phenotype, which is sparked by AMPK (AMP-activated protein kinase) activation. Therapeutic agonists, like Metformin and AICAR (5-Aminoimidazole-4-carboxamide ribonucleotide), directly upregulate AMPK activity, increasing PGC-1α (Peroxisome proliferator-activated receptor-gamma coactivator 1-alpha), OXPHOS (oxidative phosphorylation), FAO (fatty acid oxidation), and SIRT3 (Sirtuin 3). Other therapeutic agonists, like Resveratrol, can further enhance PGC-1α activity. On the other hand, the red pathway exemplifies the M1-like (pro-inflammatory) phenotype, which is dominated by mTORC1 (mammalian target of rapamycin complex 1) and HIF-1α (Hypoxia-inducible factor 1-alpha) and promotes glycolysis, as well as production of pro-inflammatory cytokines. Therapeutically, approaches that target this axis involve the inhibitors of the mTORC1 complex (e.g., Rapamycin) that can directly downregulate the activity of the mTORC1 complex, thus decreasing the levels of HIF-1α and the subsequent effects that they have. Moreover, the stabilization of the HIF-1α protein by succinate and the ROS (reactive oxygen species) constitutes another site that can be therapeutically intervened; the ROS scavenger blockers can limit the extent to this stabilization takes place. (Dashed green line resembles inhibition process)

AICAR and metformin are two extremely promising drugs that can direct microglia towards neuroprotective characteristics [Figure 4]. The cell’s principal energy sensor, AMPK, is triggered by changes in the AMP: ATP ratio, which indicates cellular energy stress (Hardie 2008). In addition to its usual function in maintaining metabolic balance, AMPK is an important modulator of immune cell activity that also affects the inflammatory response and pathways involved in cell survival. According to a wealth of research, metformin-induced AMPK activation in microglia causes a change in phenotype from pro-inflammatory (M1-like) to anti-inflammatory/pro-resolving (M2-like) (AlRuwaili et al. 2025). Mechanistically, metformin inhibits key pro-inflammatory signaling pathways by activating AMPK. Toxic cytokines like TNF-α and IL-1β are produced less frequently when they directly inhibit NF-κB activity, a transcriptional master regulator of inflammatory genes (O’Neill and Grahame Hardie 2013).

Furthermore, by blocking its upstream activators or encouraging its destruction, active AMPK directly inhibits the activation and assembly of the NLRP3 inflammasome, a multimeric protein complex that plays a key role in neuroinflammation (Al-Kuraishy et al. 2024b). This significantly reduces the release of mature IL-1β and IL-18, two powerful neurodegenerative inducers. Metformin-evoked AMPK activation not only reduces inflammation but also increases microglial phagocytic activity, which is essential for the removal of pathologic protein aggregates like alpha-synuclein in PD and Aβ in AD (Alrouji et al. 2024a). This increased clearance is frequently attributed to AMPK’s stimulation of lysosomal biogenesis and autophagy, which will enable effective cellular waste elimination (Ali et al. 2023). Furthermore, AMPK activation causes microglia to undergo a metabolic reprogramming, shifting their dependence from wasteful glycolysis to efficient OXPHOS and FAO (Al-Kuraishy et al. 2024c). A steady energy supply required for long-term homeostatic processes and the synthesis of neurotrophic factors, which support neuronal survival and synaptic health, is ensured by such metabolic reprogramming (Agostini et al. 2022). Research has demonstrated that metformin treatment can decrease microglial activation, decrease neuroinflammation, enhance mitochondrial function, and ultimately improve cognitive and motor recovery in a variety of preclinical models of neurodegenerative diseases, such as AD, PD, and ischemic stroke (Al-Kuraishy et al. 2025). For example, via modifying microglial responses, metformin has been demonstrated to at least partially diminish Aβ plaques and tau hyperphosphorylation in AD models (Al-Kuraishy et al. 2023a). In PD models, it prevents α-synuclein fibrillation and dopaminergic neuronal degeneration (Alrouji et al. 2024a). With ongoing clinical trials evaluating its neuroprotective effects, metformin is a particularly intriguing pharmacological candidate to repurpose in neurodegenerative illnesses due to its well-established safety profile and extensive clinical experience in the management of type 2 diabetes (Alrouji et al. 2023). Another well-known AMPK activator, AICAR (5-aminoimidazole-4-carboxamide ribonucleotide), also causes anti-inflammatory effects in microglia through AMPK-dependent processes, confirming AMPK as a therapeutic target for controlling microglial metabolism (Łabuzek et al. 2010).

The oral medication metformin, whose activation of AMPK is used to reduce neuroinflammation, is a major focus of the clinical translation of AMPK activators. In order to improve neuroprotection and remyelination, metformin is presently being studied in a number of clinical trials for neurodegenerative illnesses, most notably MS (e.g., NCT05893225) (De Keersmaecker et al. 2024). Although it is logistically difficult to directly examine AMPK-mediated metabolic changes in living human microglia, scientists can monitor the drug’s effects indirectly by using indicators of myeloid and glial cell activation found in cerebrospinal fluid (CSF). The idea that metabolic reprogramming is a druggable target in human neuroinflammation is supported by the argument that successful AMPK activation should be represented by a change away from the pro-inflammatory hallmark of activated cells detectable in the CSF (De Keersmaecker et al. 2024).

The greatest risk to the activators of AMPK is the difficulty of achieving an effective brain penetrance and microglial selectivity. While drugs like Metformin possess good CNS activity, direct BBB penetration is often low, and significant systemic doses are required that increase the risk of peripheral off-target activities such as hypoglycemia and lactic acidosis (Cordos et al. 2025). AMPK is a pan-tissue energy sensor; therefore, selective activation in microglia without affecting neurons and astrocytes is difficult. Subsequent research will have to focus on developing novel, brain-penetrant AMPK agonists or employing CNS-targeted delivery methods (e.g., nanocarriers or focused ultrasound) in order to maximize microglial activation with the fewest systemic metabolic tradeoffs (Cordos et al. 2025).

AMPK activators are a cornerstone of therapeutic approaches against microglial metabolism because of the pleiotropic effect of AMPK activation on microglial function, including improved clearance, anti-inflammation, and metabolic regulation.

### mTOR inhibitors

Another important class of pharmaceuticals that significantly affects microglial activation, metabolism, and autophagy is rapamycin **[**Figure 4**].**. mTOR is an essential growth factor and nutrition sensor that controls cell growth, proliferation, and protein synthesis in response to varying degrees of mixed inputs from a wide range of upstream signals. Interestingly, it also has strong autophagy inhibitory properties (Saxton and Sabatini 2017). Rapamycin primarily targets mTORC1, one of the two complexes of mTOR, mTORC1 and mTORC2. Autophagy is strongly stimulated, and anabolic activities are often suppressed when rapamycin inhibits mTORC1 (Saxton and Sabatini 2017). In pro-inflammatory (M1-like) microglia, mTORC1 is generally quite active in microglial metabolism, supporting the glycolytic shift and facilitating the rapid production of pro-inflammatory mediators [24]. Rapamycin can suppress this pro-inflammatory metabolic state by blocking mTORC1, which causes microglia to shift from glycolysis to an OXPHOS-dependent metabolism that is indicative of pro-resolving and anti-inflammatory phenotypes (Yang et al. 2021; Xu et al. 2023). This metabolic shift mitigates neuroinflammation by lowering the generation and secretion of pro-inflammatory cytokines (such as TNF-α, IL-1β) and ROS (Al-Kuraishy et al. 2023). It is noteworthy that rapamycin’s strong autophagy-inducing action is especially important for microglial function during neurodegeneration. To eliminate disease-causing protein aggregates like Aβ and α-synuclein, damaged organelles (particularly mitochondria), and cellular debris, microglia depend on effective autophagy and lysosomal activities (Ali et al. 2023).

Rapamycin reduces the inflammatory burden and creates a more enriched microenvironment by assisting microglia in more effectively clearing such toxic aggregates through the restoration and enhancement of autophagic flux (Al-Kuraishy et al. 2023b). Rapamycin treatment has been shown to decrease neuropathology, enhance cognitive function, and prolong lifespan in a variety of preclinical models of AD, PD, and Huntington’s disease. For instance, by improving the removal of misfolded proteins, rapamycin improved motor impairments in PD models and decreased tau and Aβ pathology in AD models (Dumont and Su 1995; Al-Kuraishy et al. 2023). Although rapamycin has therapeutic potential, its systemic immunosuppressive action, which stems from its all-encompassing effect on immune cells, presents a major obstacle to its use in treating chronic neurological illnesses (Dumont and Su 1995).

The mTOR inhibitor rapamycin, also known as sirolimus, is widely utilized in clinical and human translational investigations, especially as an immunosuppressant and in several cancer trials, demonstrating its proven tolerability and BBB-crossing capabilities (Seto 2012). In contrast to rodent models, research on human microglia, such as that conducted with the HMC3 cell line, reveals intricate and occasionally contradicting effects (Dello Russo et al. 2018). For example, rapamycin may have the opposite effect of its assumed neuroprotective function by causing human microglia to express more pro-inflammatory cytokines, such as IL-6 (Dello Russo et al. 2018). The direct microglial metabolic signature regulated by mTOR inhibition in living patients with neurodegenerative diseases still needs specialized clinical trials to completely close the gap between preclinical mechanism and human efficacy, even though CSF analysis is used to identify markers linked to inflammation.

Immunosuppression, particularly with Rapamycin’s prolonged administration, is the primary translational challenge for mTOR inhibitors. In addition, mTORC1 inhibition can also lead to more severe metabolic tradeoffs, including insulin resistance and hyperlipidemia (Marafie et al. 2024). Target selectivity optimization towards mTORC1 over mTORC2 is required because only mTORC1 is commonly a target for anti-inflammatory use. Novel, non-immunosuppressive rapalogs or formulations engineered for brain-cell selectivity will have to be created to capitalize on their anti-neuroinflammatory potential while maintaining systemic immune function.

In order to maximize its positive effects on microglial metabolism and autophagy and reduce systemic side effects, future research will concentrate on creating brain-permeant, mTOR-selective inhibitors or localized CNS delivery strategies.

### Ketone body supplementation

Ketone bodies, particularly those containing β-hydroxybutyrate (BHB), provide a direct and effective metabolic intervention to reduce neuroinflammation and modify microglial activation. Under low-glucose circumstances (such as fasting or a ketogenic diet), the liver produces ketones, primarily BHB and acetoacetate, as an alternative energy source (Shahpasand et al. 2024). They are a very efficient source of fuel for neurons and glial cells, including microglia, and are easily transported across the BBB (Shahpasand et al. 2024). BHB is a signaling molecule that has built-in anti-inflammatory and neuroprotective qualities in addition to being an energy source. The direct suppression of the NLRP3 inflammasome, a key element of the innate immunity apparatus that, when activated, results in the production of highly active pro-inflammatory cytokines like IL-1β, is one of the important ways that BHB controls microglial metabolism and activity (Blevins et al. 2022). BHB accomplishes this suppression by blocking caspase-1 activation, which stops mature IL-1β and IL-18 from being released through proteolytic cleavage (Blevins et al. 2022). In order to reduce the neuroinflammatory cascade that is characteristic of many neurodegenerative disorders, this in vitro anti-inflammatory impact is essential. Additionally, a metabolic change to OXPHOS, with a bias towards a lower inflammatory, pro-resolving phenotype, can be triggered by BHB metabolism in microglia (Blevins et al. 2022). Ketone body supplementation has shown promise as a treatment in several preclinical models of neurological diseases. By modifying microglial function and lowering neuroinflammation, BHB improved mitochondrial function, decreased Aβ pathology, and improved cognitive impairments in AD models (Jang et al. 2024). Ketone bodies have also been demonstrated to have anticonvulsant effects in models of epilepsy; these effects are believed to be mediated by some of their effects on microglial activity and neuronal metabolism, which lower inflammation and hyperexcitability (Jang et al. 2024). Furthermore, by blocking histone deacetylases (HDACs), BHB may function as an epigenetic regulator, influencing gene expression patterns in microglia in the direction of a more advantageous phenotype (Delcuve et al. 2012). Ketone bodies are a potent therapeutic approach for reducing neuroinflammation and enhancing neuroprotection in neurodegenerative diseases because of their capacity to supply a novel fuel source, directly inhibit important inflammatory pathways, and epigenetically modify microglial function.

A promising metabolic treatment for neurodegenerative diseases is supplementing with ketone bodies, primarily β-hydroxybutyrate (BHB). In human cellular models, BHB has been shown to modify microglial metabolism and prevent inflammation and phagocytic dysfunction caused by Aβ oligomers, a key pathology in AD (Jin et al. 2023). To determine precisely how exogenous ketone esters increase BHB concentrations in human subjects’ CSF, a crucial step in demonstrating target engagement of the CNS, they are being used in specialized clinical trials (Jin et al. 2023). This is essential for translating BHB’s anti-inflammatory effects on microglia that have been seen in vitro into useful neuroprotective treatments for patients.

Translational feasibility of ketogenic approaches (diet or exogenous ketones) is constrained by compliance and metabolic tradeoff factors (Na et al. 2025). Patient compliance with a strict KD over the long term is challenging, and the diet per se is risky, such as dyslipidemia, kidney stones, and gastrointestinal upset. Exogenous ketone supplements provide less stable metabolic control compared to the KD and can induce gastrointestinal discomfort. Inadequate control of the narrow therapeutic window (plasma β-hydroxybutyrate concentrations) and off-target action in non-microglial cells necessitate the creation of more targeted delivery vehicles or ketone esters to deliver CNS efficacy and patient safety (Na et al. 2025).

### Lipid metabolism

A useful therapeutic target outside of the conventional anti-inflammatory strategy is microglial lipid metabolism. As extremely active phagocytic cells, microglia play a crucial role in lipid processing, especially in neurodegenerative diseases when they come into contact with lipid-rich myelin debris or dying neuronal membranes (Yang et al. 2016). Lipid droplets (LDs) are accumulated by microglia in chronic inflammatory situations or pathological accumulations (e.g., Aβ in AD). These LDs are dynamic organelles with the capacity to produce inflammatory lipids and are frequently linked to pro-inflammatory, defective microglial function, as well as compromised phagocytosis (Li et al. 2024b). Lipotoxicity, mitochondrial dysfunction, and a diminished ability to remove abnormal aggregates are all possible outcomes of this lipid accumulation, which could fuel neuroinflammation and hasten neurodegeneration (Li et al. 2024b). Pharmacologic modulators of microglial lipid metabolism are therefore of therapeutic interest, especially those that promote the breakdown of LDs or activate FAO. The leading candidates are PPAR agonists, especially PPAR-α and PPAR-γ. The FAO and genes linked to lipid catabolism are known to be regulated by these nuclear hormone receptors (Titus et al. 2024). Drugs can improve mitochondrial activity, decrease lipid buildup, increase FAO in microglia, and initiate a change towards an anti-inflammatory phenotype by activating PPARs (Titus et al. 2024). For example, in AD models, PPAR-γ agonists have been used to lower neuroinflammation and microglial activation (Titus et al. 2024).

Microglial function may be altered by tactics that improve the internalization and utilization of lipids that are introduced exogenously or stabilize endogenous lipid production pathways. For instance, stimulating microglia to a more pro-resolving or homeostatic phenotype will improve their capacity to remove cellular waste and reduce inflammation by increasing the absorption of particular fatty acids or encouraging their optimum oxidation (Traetta et al. 2025). Research into microglial lipid metabolism is crucial because there is mounting evidence that dysregulated lipid processing contributes to neuroinflammation and dementia. Lipid-laden microglia that develop triglyceride-rich lipid droplets are a major pathogenic characteristic of human tissue from AD and PD patients. These microglia frequently have a pro-inflammatory, dysfunctional behavior. Clinical trials that target microglial lipid homeostasis, such as treatments that regulate important lipid-associated genes like APOE and TREM2, are being driven by these discoveries (Sprenger et al. 2025). Additionally, a non-invasive method being used in trials to track the metabolic status of microglia and the effectiveness of treatments in the CNS is the analysis of the CSF for lipid metabolites and inflammatory markers (Sprenger et al. 2025). The next stages for this extremely promising treatment approach are to identify the particular mechanisms by which lipid metabolism is altered in various disorders and to build highly selective modulators for these processes in microglia.

Their PPAR agonists face the double challenge of target specificity and extreme off-target toxicity (Rubenstrunk et al. 2007). PPAR subtypes are present in many tissues, and current non-selective agonists, such as the PPARγ agonist Pioglitazone, are linked to severe peripheral side effects, including edema, weight gain, and congestive heart failure, that limit their use on a long-term basis in vulnerable patient populations (Rubenstrunk et al. 2007). For successful microglial therapy, next-generation PPAR agonists must be designed with improved microglial selectivity for PPARγ and deep brain penetrance with an improved safety profile that is decoupled from their systemic metabolic effects (Rubenstrunk et al. 2007).

### HIF-1α modulators

A novel strategy to regulate the harmful glycolytic changes seen in microglia during neuroinflammation is provided by HIF-1α modulators **[**Figure 4**].**. By encouraging glycolysis and preventing oxidative phosphorylation, the master transcriptional regulator HIF-1α coordinates the cell’s hypoxic response. Although low oxygen is the primary cause, inflammatory stimuli (such as LPS, pro-inflammatory cytokines, and ROS) stabilize HIF-1α at normoxia (Shen et al. 2024). The pro-inflammatory (M1-like) state of microglia is characterized by stabilization, which promotes metabolic reprogramming to aerobic glycolysis (Shen et al. 2024). This reliance on glycolysis provides instantaneous ATP to support the strong production of ROS and pro-inflammatory cytokines (such as TNF-α and IL-1β), which leads to neurotoxicity and facilitates neuronal death in conditions including AD, PD, and stroke (Shen et al. 2024). Pharmacological inhibitors of HIF-1α activity, such as certain tiny compounds that prevent it from stabilizing or binding to DNA, may therefore be a great way to prevent or correct this pathologic glycolytic shift in microglia (Zhang et al. 2025). Such modulators may prevent the synthesis of inflammatory mediators and change microglial metabolism to a more oxidative, cost-effective state, thereby promoting an anti-inflammatory and pro-resolving phenotype by blocking HIF-1α-mediated glycolysis (Kim and Lee 2025). Inhibition of HIF-1α has been demonstrated in preclinical models of neuroinflammation to potentially decrease microglial activation, decrease the expression of inflammatory cytokines, and increase neuronal damage (Dong et al. 2024). By altering the microglial response, inhibition of HIF-1α has been demonstrated to decrease infarct volume and improve neurological recovery in models of cerebral ischemia (Dong et al. 2024).

The transcription factor HIF-1α is a master regulator of the pro-inflammatory (M1-like) phenotype in activated microglia, which makes HIF-1α modulators promising targets for neuroinflammation therapy (Dong et al. 2025). Studies on human tissue have revealed that disorders such as AD are associated with a particular subset of inflammatory-angiogenic microglia that expresses more HIF-1α. The assessment of downstream metabolites controlled by HIF-1α in CSF is being investigated as a possible biomarker for the advancement of the disease and the effectiveness of treatment (Dong et al. 2025). While most direct clinical trials targeting microglial HIF-1α are still in the preclinical and early stages of development, a promising method to therapeutically rewire microglial metabolism to a less neurotoxic state is to modulate this pathway, frequently with prolyl hydroxylase domain inhibitors.

Inhibition of HIF-1α entails an extremely high risk of off-target in numerous tissues and general cellular toxicity because it is a master transcription factor that controls basic oxygen sensing and cell survival across all cell types (Zhang et al. 2025). Non-selective inhibition or stabilization can impair critical functions like anti-cancer protection or systemic EPO regulation. The greatest unknown is how to perform exact, cell-type-selective modulation. Modulators with exceptional target selectivity and delivery technologies, such as nanocarrier or viral vector-mediated microglial-specific targeting, to avoid diffuse systemic or neuronal toxicities from non-discriminatory perturbation of cellular oxygen sensing are required for translational success (Zhang et al. 2025).

Therefore, the challenge is to create modulators that can precisely control HIF-1α activity, supporting therapeutic acute responses while suppressing harmful, chronic glycolytic shift in microglia. This approach differs from general anti-inflammatory medications in that it is a very specific treatment to rewire microglial metabolism and decrease neuroinflammation.

### Gut microbiome modulation

By adjusting the complex gut-brain axis, gut microbiota modulation is a rapidly developing and very successful indirect medicinal approach to control microglial metabolism and CNS inflammation. A diverse range of metabolites, including SCFAs like butyrate, propionate, and acetate, are produced by the gut microbiota, a large and diverse community of microorganisms that live in the intestinal tract (O’Riordan et al. 2022). These SCFAs have direct effects on brain function and microglial activation by interacting with immune cells, the vagus nerve, and the BBB (O’Riordan et al. 2022). In addition to providing microglia with an energy substrate, butyrate is a strong anti-inflammatory chemical that has been demonstrated to directly impact microglial phenotype by blocking HDACs, which modifies gene expression patterns to a more favorable, anti-inflammatory degree (Cao et al. 2025b). Increased gut permeability (also known as “leaky gut”) and neurodegenerative diseases (such as AD and PD) are increasingly associated with dysbiosis, or an imbalance of the gut microbiota (Cao et al. 2025b). Because of this weakened gut barrier, inflammatory mediators and bacterial products (such as lipopolysaccharide, or LPS) can enter the bloodstream and cause systemic inflammation, which can then trigger or worsen neuroinflammation in the CNS (Di Vincenzo et al. 2024). Probiotics, which are live, non-pathogenic bacteria, and prebiotics, which are unabsorbable carbohydrates that specifically promote the growth and/or activity of the beneficial gut microbes, are therapeutic interventions for the restoration of gut microbial balance (Di Vincenzo et al. 2024). By promoting the synthesis of advantageous SCFAs, reducing intestinal inflammation, and enhancing gut barrier integrity, these therapies can indirectly suppress neuroinflammation and alter the metabolic phenotypes of microglia (Cheng et al. 2024).

In preclinical models, faecal microbiota transplantation (FMT), a more invasive procedure that involves directly implanting faecal material from a healthy donor into a patient, is being investigated for its potential to restore a healthy microbiome and to reduce neuroinflammation. In certain neurodegenerative conditions, FMT has shown promising initial results (Hamamah et al. 2022). Gut microbiome modulation is a new and simple therapeutic approach that indirectly affects microglial function and metabolism, resulting in neuroprotection in a variety of CNS disorders by restoring gut eubiosis, lowering systemic inflammation, and stimulating the synthesis of neuroactive metabolites (Che Mohd Nassir et al. 2025).

Through microbial metabolites, gut microbiome modification provides a way to affect microglial activation through the gut-brain axis. Since the amounts of microbial-derived SCFAs in human tissue and CSF are correlated with indicators of microglial activation and the course of neurodegenerative diseases, including PD and AD, research on people focuses on detecting these SCFAs (Loh et al. 2024). Interventions like faecal microbiota transplantation, probiotics, and prebiotics are being studied in clinical trials. With more and more data proving its effectiveness as a unique therapeutic approach, these trials seek to re-establish a healthy microbial balance in order to reduce systemic and central neuroinflammation and maybe enhance cognitive and behavioral results (Sahle et al. 2024).

Microbiome therapeutics like Fecal Microbiota Transplantation and SCFA delivery are hampered by issues of reproducibility and CNS-targeted delivery. FMT efficacy is inherently variable and non-standardizable. In the case of SCFAs, the most significant scientific uncertainty is the degree of penetrance into the brain of different short-chain fatty acids and their particular downstream microglial targets (Karimi et al. 2024). Strategies will have to surmount the poor compliance of restrictive diets or the non-sterile, multifaceted nature of FMT. The future will rely on precise delivery of a standardized combination of beneficial, BBB-permeating metabolites or constructed microbes (Karimi et al. 2024).

## Limitations and future perspectives

Although there have been positive developments in our knowledge of microglial metabolic reprogramming and its potential for treatment, a number of important obstacles must be overcome before these developments may lead to successful clinical treatments for neurodegenerative illnesses. The complexity and diversity of microglial states in living things are one of the biggest drawbacks. Despite being a helpful conceptual model, the M1-LIKE/M2-LIKE paradigm overstates a range of microglial phenotypes, each of which has distinct metabolic requirements and functional outputs. In order to replicate the complex metabolic changes and dynamic interactions of microglia within the heterochromatic complexity of the human brain, particularly over the long, progressive duration of neurodegenerative diseases, available studies have typically used in vitro systems or simplified in vivo models of injury. Furthermore, it is difficult for pharmacologic modulators to accurately and specifically target microglial metabolic pathways in the brain. Although promising, systemic drug treatment with medications like rapamycin or metformin will have off-target effects in peripheral tissues. As a result, dosage and potentially new delivery technologies (such as targeted ultrasound, nanocarriers, or gene therapy) must be carefully considered to achieve adequate brain penetration and minimize systemic side effects. Additionally, the effectiveness and safety of long-term microglial metabolism modification are still unclear. Chronic changes may arise and result in unfavorable outcomes, such as impaired homeostatic functions (e.g., synaptic refinement, waste elimination) or increased susceptibility to infections, even while acute metabolic changes are advantageous. In addition, the heterogeneity in metabolic patterns, genetic backgrounds, and gut microbiomes among neurodegenerative patients complicates the use of universal therapeutic approaches. A “one-size-fits-all” strategy would not be ideal, and personalized medicine is emphasized herein.

Some of the most important features will encourage more innovation in this area in the future. The need for more advanced in vivo instruments to more accurately depict the metabolic states of microglia in real time within living brains is at the top of this list. To understand the complex metabolic landscape of microglial subpopulations in disease, these include the development of sophisticated metabolic imaging techniques (e.g., metabolic PET or MRI), genetically encoded metabolic sensors in microglia, and single-cell multi-omics approaches (e.g., single-cell RNA sequencing combined with metabolomics or fluxomics). To enable highly focused therapies, future studies must concentrate on finding certain metabolic deficiencies within particular microglial phenotypes that promote disease. For example, pro-inflammatory microglia’s glycolysis is selectively inhibited without compromising the metabolic flexibility needed for resolution. Finding novel pharmaceutical targets that have enhanced brain penetration and microglial selectivity, possibly focusing on transporters or enzymes unique to microglial metabolism, will be essential. Furthermore, there is great potential for combination treatment with pharmacological modulators and nutritional strategies (such as calorie restriction or ketogenic diets) or gut microbiome modulation (such as using probiotics or prebiotics) to target microglial metabolism and inhibit neuroinflammation in parallel mechanisms.

Future innovation in this field will be stimulated by some of the most significant qualities. The requirement for sophisticated tools to precisely represent the metabolic states of microglia at the single-cell level is critical in order to overcome the drawbacks of bulk analysis and the M1/M2 simplification. This includes the creation of genetically encoded metabolic sensors and advanced metabolic imaging methods (such as metabolic PET or MRI). Importantly, it will be crucial to integrate single-cell multi-omics techniques, including single-cell RNA sequencing (scRNA-seq) with single-cell fluxomics or metabolomics. By defining distinct metabolic signatures for particular disease-associated microglial states, this high-resolution mapping can pinpoint the exact enzymatic and transporter requirements of pathogenic subpopulations. Precision therapy development is based on this fine-grained knowledge. Future research must focus on identifying metabolic weaknesses in certain microglial phenotypes that contribute to disease, such as blocking pro-inflammatory microglia’s enhanced glycolysis specifically, without sacrificing the resolution-related flexibility. Finding new pharmacological treatments with improved brain penetration and microglial-selective action, possibly concentrating on particular microglial transporters or enzymes, will be necessary for this shift to precision targeting. Additionally, combination therapy has significant promise for targeting microglial metabolism and inhibiting neuroinflammation through parallel, customized processes by utilizing pharmacological modulators in conjunction with dietary or gut microbiome manipulation (e.g., probiotics or prebiotics).

Lastly, patient categorization, therapy efficacy evaluation, and the development of individualized treatment plans will all depend on the discovery of reliable peripheral or CSF metabolic indicators that faithfully represent CNS microglial metabolic states. Microglial metabolic reprogramming presents a promising and potentially revolutionary platform for the development of disease-modifying treatments for neurodegenerative diseases by overcoming these obstacles and adopting innovative research approaches [Table 2].

**Table 2:** Translational status of microglial metabolic interventions

Intervention	Mechanism	Stage of Evidence	Key Translational Barrier(s)	References
Metformin	Activates AMPK, favoring M2-like OXPHOS/FAO	Preclinical (Strong), Clinical Trials (Phase II/III, repurposed)	Off-target systemic effects; achieving therapeutic brain concentration; determining optimal dosage for brain inflammation vs. peripheral metabolism.	(Abdi et al. 2021)
Rapamycin	Inhibits mTOR, promoting autophagy and M2-like shift	Preclinical (Strong), Clinical Trials (Limited, repurposed)	High systemic toxicity/immunosuppression risk; poor blood-brain barrier (BBB) penetration; need for microglial-specific delivery.	(Gonzales et al. 2025)
Ketogenic Diets/Ketone Bodies	Provides alternative fuel source (BHB), modulating G-protein receptors (GPRs)	Preclinical (Strong), Human Studies (Observational, Small Clinical Trials)	Patient adherence and safety profile for long-term use; high variability in patient metabolic response; need for rigorous, large-scale randomized controlled trials (RCTs).	(Vieira et al. 2025)
Gut Microbiome Modulation (Probiotics/SCFAs)	Generates Short-Chain Fatty Acids (SCFAs) (e.g., Butyrate) that signal through GPRs and reinforce the BBB	Preclinical (Mechanistic), Human Studies (Early Clinical Trials)	Causal vs. correlative relationship in humans; high inter-individual variability in gut microbiota; delivery and stability of specific beneficial strains; low CSF SCFA concentration.	(Blaak et al. 2020)
Specific Glycolysis Inhibitors (e.g., 2-DG analogs)	Inhibits HK/PFK to block the M1-like glycolytic switch	Preclinical (Mechanistic, in vitro/animal models)	High risk of energy-depletion off-target effects in neurons and other brain cells; lack of microglial-selective targeting mechanism.	(Cheng et al. 2021)

## Conclusion

Our knowledge of the function of microglia in the CNS has been completely transformed by the emerging science of immunometabolism. According to this review, microglial metabolic rewiring is a crucial determinant of their functional phenotype, either promoting neuroinflammation and leading to neurodegeneration or initiating neuroprotection and repair. We described how different metabolic changes underlie particular microglial functions in both healthy individuals and a range of neurodegenerative diseases, such as AD, PD, MS, stroke, and TBI. These changes range from pro-inflammatory glycolysis to anti-inflammatory oxidative phosphorylation and fatty acid oxidation.

With several points of therapeutic intervention, these transitions are guided by complex regulatory networks that include AMPK, mTOR, and HIF-1α. Promising approaches to rewire microglia towards a healthy phenotype include nutritional interventions like ketogenic diets and gut microbiome modification, as well as pharmaceutical modulators like metformin and rapamycin. Microglial metabolic regulation is a new frontier, although precise targeting and long-term safety are still challenges. By utilizing these findings, future studies may pave the way for novel, disease-modifying treatments that improve microglial homeostasis, reduce neuroinflammation, and eventually slow down or prevent catastrophic neurodegenerative illnesses.

## Data Availability

No datasets were generated or analysed during the current study.

## Abbreviations

AD: Alzheimer’s Disease
AMPK: AMP-activated protein kinase
ATP: Adenosine triphosphate
Aβ: Amyloid-beta
BBB: Blood-brain barrier
BDNF: Brain-derived neurotrophic factor
BHB: β-hydroxybutyrate
CNS: Central nervous system
CPT1: Carnitine palmitoyl transferase 1
CSF: Cerebrospinal fluid
DAMP: Danger-associated molecular pattern
DHA: Docosahexaenoic acid
EPA: Eicosapentaenoic acid
FAO: Fatty acid oxidation
FMT: Faecal microbiota transplantation
GLUT1: Glucose transporter 1
HIF-1α: Hypoxia-inducible factor 1-alpha
IL-1β: Interleukin-1 beta
IL-10: Interleukin-10
KD: Ketogenic diet
LPS: Lipopolysaccharide
LD: Lipid droplet
M1-like: Pro-inflammatory microglial phenotype
M2-like: Anti-inflammatory/resolving microglial phenotype
MS: Multiple Sclerosis
mTOR: Mammalian target of rapamycin
mTORC1: mTOR Complex 1
mTORC2: mTOR Complex 2
NF-κB: Nuclear factor kappa-light-chain-enhancer of activated B cells
NLRP3: NLR family pyrin domain containing 3 (inflammasome)
OXPHOS: Oxidative phosphorylation
PAMP: Pathogen-associated molecular pattern
PD: Parkinson’s Disease
PDH: Pyruvate dehydrogenase
PPAR-γ: Peroxisome proliferator-activated receptor gamma
ROS: Reactive oxygen species
SCFA: Short-chain fatty acid
SPM: Specialized pro-resolving mediator
TBI: Traumatic brain injury
TGF-β: Transforming growth factor beta
TNF-α: Tumor necrosis factor-alpha
TCA: Tricarboxylic acid (cycle)
VHL: Von Hippel-Lindau (tumor suppressor protein)
